# Bone Marrow Mesenchymal Stromal Cells and Their Derived Extracellular Vesicles Protect Pancreatic Beta‐TC‐6 Cells From Hypoxia‐Induced Injury via miR‐539‐3p‐Mediated Downregulation of CD36 Expression

**DOI:** 10.1155/sci/6616986

**Published:** 2026-01-21

**Authors:** Na Lin, Yaoyao Liang, Minying Tang, Fei Liu, Liuyan Chen, Lvying Wu, Yunfeng Fu, Zhuoyu Li, Lingfeng Zhu, Jin Chen

**Affiliations:** ^1^ Minimally Invasive Urology and Translational Medicine Center, Fuzhou First General Hospital Affiliated with Fujian Medical University, Fuzhou, Fujian Province, China, fjmu.edu.cn; ^2^ Institute of Clinical Medicine, The Second Affiliated Hospital of Hainan Medical University, Haikou, Hainan Province, China, hainmc.edu.cn; ^3^ Fujian Provincial Key Laboratory of Transplant Biology, The 900th Hospital, Xiamen University, Fuzhou, Fujian Province, China, xmu.edu.cn; ^4^ Fujian Provincial Stem Cell Application Engineering Technology Research Center, Fuzhou General Clinical Medical School, Fujian Medical University, Fuzhou, Fujian Province, China, fjmu.edu.cn

**Keywords:** beta-TC-6 cells, bone marrow mesenchymal stromal cell (BMSC), CD36, extracellular vesicle, hypoxia, miR-539-3p

## Abstract

Bone marrow mesenchymal stromal cells (BMSCs) have been shown to enhance the function of pancreatic beta‐cells under hypoxic conditions. However, the precise mechanisms underlying this protective effect remain elusive. In this study, we established a hypoxic beta‐cell model using murine pancreatic beta‐TC‐6 cells to investigate the protective effect and mechanism of BMSCs and their secreted extracellular vesicles (BMSC‐EVs) on hypoxic β cells. Our findings reveal that coculture with BMSCs or BMSC‐EVs significantly enhances the viability and survival of hypoxic beta‐TC‐6 cells. Molecularly, hypoxic conditions trigger an upregulation of CD36 in beta‐TC‐6 cells, a response that is counteracted by BMSCs or BMSC‐EVs. Through a screening process for microRNAs (miRNAs) capable of degrading CD36 mRNA, we identified miR‐539‐3p as a potent suppressor of CD36 expression. The miR‐539‐3p mimic was found to bolster the viability of hypoxic beta‐TC‐6 cells, concurrently reducing CD36 mRNA levels by targeting its 3’ untranslated region (3’UTR). In contrast, the miR‐539‐3p inhibitor abrogates the protective effects of BMSCs and BMSC‐EVs on these cells. Additionally, knockdown of CD36 in hypoxic beta‐TC‐6 cells restores the protective function mitigated by miR‐539‐3p inhibition. In aggregate, these results suggest that BMSCs and BMSC‐EVs shield beta‐TC‐6 cells from hypoxia‐induced injury through miR‐539‐3p‐mediated downregulation of CD36, underscoring the therapeutic potential of targeting the miR‐539‐3p‐CD36 axis to enhance pancreatic beta‐cell function in diabetic patients.

## 1. Introduction

Diabetes has emerged as a significant global health challenge, with its prevalence steadily increasing over the past few decades due to factors such as aging populations and rising obesity rates [1]. By 2045, it is projected that ~783 million individuals will be living with diabetes [2]. Notably, around 90% of diabetes cases are classified as Type 2 diabetes (T2D) [3].

Insulin, a hormone produced by the beta‐cells of the pancreas, plays a crucial role in regulating blood glucose levels [4]. Dysfunction of these beta‐cells can lead to insufficient insulin production, resulting in hyperglycemia and the eventual onset of diabetes [5, 6]. The impairment of beta‐cell function is influenced by a complex interplay of genetic, metabolic, environmental, and molecular factors. Specific genetic variants in key regulators of beta‐cell function, such as TCF7L2, KCNJ11, and HHEX, have been associated with beta‐cell dysfunction [7]. Additionally, chronic hyperglycemia and prolonged exposure to elevated levels of free fatty acids contribute to beta‐cell malfunction and programmed cell death [8]. Elevated levels of reactive oxygen species (ROS) [9], endoplasmic reticulum (ER) stress [10, 11], and stimulation by pro‐inflammatory factors have also been shown to adversely affect beta‐cell function and survival [12].

Hypoxia is another significant factor contributing to beta‐cell dysfunction in diabetes [13]. It can arise from various conditions, including islet hyperplasia and inflammation [14, 15]. Hypoxia leads to increased ROS levels, heightened ER stress, and mitochondrial dysfunction within beta‐cells, impairing their insulin secretion and survival [11, 14, 16]. However, the precise mechanisms underlying hypoxia‐induced injury to beta‐cells remain incompletely understood.

Bone marrow mesenchymal stromal cells (BMSCs) exhibit multipotency, enabling them to differentiate into multiple cell lineages. Studies have shown that BMSCs can differentiate into beta‐cells in vivo and alleviate diabetic phenotypes in animal models [17, 18]. Furthermore, BMSCs promote beta‐cell regeneration through various mechanisms [19, 20]. Nevertheless, the effects of BMSCs on hypoxic beta‐cells are still not fully elucidated.

The paracrine pathway is crucial for BMSCs to exert their functions [21]. In addition to secreting cytokines, BMSCs produce and release extracellular vesicles (EVs), also known as exosomes, which can influence target cells in diverse contexts [22–24]. MSCs from other sources have been shown to secrete EVs that regulate beta‐cell function and activity. For instance, EVs derived from menstrual blood MSCs enhance beta‐cell regeneration and function in a rat model of Type 1 diabetes (T1D) through a mechanism involving the PDX1 protein [25]. Similarly, EVs from human umbilical cord‐derived MSCs have been found to reduce beta‐cell damage and improve insulin sensitivity in a rat model of T2D [26]. However, it remains unclear whether BMSC‐derived EVs can protect beta‐cells from hypoxia‐induced injury.

To address these questions, we first established a hypoxia model using beta‐TC‐6 cells. We then investigated the effects of BMSCs and their EVs on the viability, colony formation ability, and apoptosis of cocultured hypoxic beta‐TC‐6 cells. Our findings revealed that both BMSCs and their EVs significantly downregulated CD36 expression in hypoxic beta‐TC‐6 cells. Through screening for microRNAs (miRNAs) targeting CD36, we identified miR‐539‐3p as a potent repressor of CD36 expression. The miR‐539‐3p mimic was found to enhance the viability of hypoxic beta‐TC‐6 cells while effectively reducing CD36 mRNA levels by binding to its 3’ untranslated region (3’UTR). Furthermore, we explored the effects of the miR‐539‐3p mimic and inhibitor on hypoxic beta‐TC‐6 cells. Finally, we depleted CD36 in hypoxic beta‐TC‐6 cells cocultured with BMSCs or BMSCs and their secreted EVs (BMSC‐EVs) in the presence or absence of the miR‐539‐3p inhibitor, evaluating their viability, colony formation ability, and apoptosis. Our data reveal a novel mechanism through which BMSCs and their EVs exert protective effects on hypoxic beta‐TC‐6 cells.

## 2. Materials and Methods

### 2.1. Cells

Mouse BMSCs were obtained from IMMOCELL (Xiamen, China) and cultured in α‐MEM medium supplemented with 10% FBS (Gibco, USA). Beta‐TC‐6 cells were acquired from the Cell Bank of the Typical Culture Preservation Committee of Chinese Academy of Sciences (Shanghai, China) and maintained in high‐glucose DMEM medium (Gibco, USA) supplemented with 15% FBS under normoxic conditions (37°C, 5% CO_2_, 21% O_2_). To induce hypoxic coculture, the beta‐TC‐6 cells were subjected to a hypoxic environment (37°C, 2% O_2_, 5% CO_2_).

### 2.2. Plasmids and miRNA Mimics/Inhibitors

The wildtype (WT) or mutant 3’UTR of CD36 was cloned into the pmirGLO plasmid. Short hairpin RNAs (shRNAs) targeting CD36 were inserted into the pLKO.1‐TRC‐puro plasmid. The primers sequences for plasmid construction and the sequence of miRNA mimics/inhibitors are shown in Supporting Information 1: Table S1.

### 2.3. Plasmid Transfection and Virus Infection

Plasmid transfections were performed using Lipofectamine 2000 (Thermo Fisher Scientific, USA). Lentiviral particles for CD36 knockdown were obtained from Anti‐Hela Biotechnology (Xiamen, China). The multiplicity of infection (MOI) was optimized to 10 for beta‐TC‐6 cell infection using TaqMan real‐time PCR.

### 2.4. EVs Purification and Characterization

BMSCs were cultured in 10% EV‐free FBS medium for 48 h, after which the medium was harvested for EV purification. Sequential centrifugation steps were performed at 2000 *g* for 30 min, followed by 10,000 *g* for 30 min at 4°C. The supernatant was filtered through a 0.45 μm filter and centrifuged at 100,000 *g* for 60 min at 4°C. The resulting pellet was resuspended in pre‐cooled PBS, filtered through a 0.22 μm filter, centrifuged at 100,000 *g* for 60 min at 4°C again, and resuspended in 100 μL of ice‐cold PBS. For EVs quantification, aliquots were analyzed using transmission electron microscopy (TEM, JEM‐1230 electron microscope) and nanoparticle tracking analysis (NTA, NanoSight LM10 system). Western blot was used to assess the presence of EV markers CD9, CD81, and CD63. The remaining EVs were stored at −80°C for further analysis. BMSC‐EVs were quantified based on protein content. The quantification was performed using a BCA protein assay kit.

### 2.5. Coculture With BMSCs or BMSC‐EVs

Following a 24‐h adhesion period, Beta‐TC‐6 cells were co‐cultured for 48 h with either BMSCs using a transwell system (at a cell‐to‐cell ratio of 5:1) or with BMSC‐EVs at a concentration of 50 μg/mL, which was determined from prior optimization experiments. Cells were subsequently harvested for analysis.

### 2.6. Western Blot

Protein extraction and quantification were carried out using a BCA kit (Tiangen). Proteins were denatured, separated on SDS‐PAGE gels, and transferred to PVDF membranes. After blocking with 5% nonfat milk, membranes were incubated with primary antibodies overnight at 4°C, followed by incubation with secondary antibodies for 2 h at room temperature (RT). ECL kit (Tiangen) was used to visualize signals, which were recorded on a ChemiDoc device (BioRad, USA) and quantified using Image J (NIH, USA). A list of antibodies is provided in Supporting Information 1: Table S2.

### 2.7. Quantitative Real‐Time Polymerase Chain Reaction (qPCR)

Total RNA from BMSCs and their EVs was extracted using Trizol reagent (Thermo Fisher Scientific, USA). cDNA synthesis was performed using the HiScript II QRT SuperMix (Vazyme, China). qPCR was conducted using the ChamQ SYBR Color qPCR Master Mix (Vazyme) on an ABI 7500 machine (Applied Biosystems, USA). Gene expression was calculated using the 2^−ΔΔCt^ method and normalized to 18S rRNA or U6. Primer sequences are listed in Supporting Information 1: Table S3.

### 2.8. Cell Viability Assay

Beta‐TC‐6 cell viability was assessed using the cell counting kit (CCK‐8) (Monmouth Junction, NY, USA). Briefly, Cells were seeded into 96‐well plates at a density of 10,000 cells/well and treated as indicated. After incubation, 10 μL of CCK‐8 solution was added, and cells were incubated for 2–3 h at 37°C. The OD value at 450 nm (OD_450_) was measured using a microplate reader.

### 2.9. Apoptosis

Apoptosis was evaluated using the Annexin V FITC Kit (Beyotime Biotechnology, China). Briefly, cells were harvested, resuspended, and incubated with PI/Annexin V‐FITC working solution in a light‐protected environment for 15 min at RT. Subsequently, the samples were analyzed using a NovoCyte FACS cytometer (Agilent).

### 2.10. TUNEL Assay

TUNEL assay was performed using the CoraLite 488 Plus TUNEL apoptosis detection kit (Proteintech, China). Collect the cells, wash the cells twice with PBS, then the cells were fixed with 4% paraformaldehyde solution at 4°C for 30 min, washed twice with PBS, permeabilized using PBS solution containing 0.2% Triton X‐100, left at RT for 20 min, and washed twice again with PBS. Add 50 μL of the TUNEL reaction mixture (containing TdT enzyme) to the sample, and incubate at 37°C in the dark for 60 min. Discard the reaction solution and washed twice with PBS, each time for 5 min. Then, washed with the buffer prepared in PBS containing 0.1% Triton X‐100 and 5 mg/mL BSA. 2 μg/mL DAPI staining solution was added and incubated in the dark at RT for 10 min. After staining, washed with PBS three times, each time for 5 min, and finally, observed under the microscope.

### 2.11. Carboxyfluorescein Succinimidyl Ester (CFSE) Tracing

EV suspension was labeled with CFSE dye (Yeasen, China) and filtered using 100 kDa MWCO centrifugal filters (Millipore, USA). The labeled EVs were cultured with cells for 0, 24, or 48 h at 37°C. Cells were fixed with 4% paraformaldehyde and imaged using an Axio Imager M2 microscope (Carl Zeiss AG, Germany).

### 2.12. Colony Formation Assay

Beta‐TC‐6 cells were seeded in 6 cm culture dishes and cultivated for 2 weeks with medium changes every 3 days. After incubation, cells were fixed, stained with Giemsa, and imaged. Colony numbers were quantified using ImageJ.

### 2.13. Dual Luciferase Assay

The Dual‐Glo Luciferase Assay System (Promega, USA) was employed to evaluate the activity of luciferases linked with the WT or mutant 3’UTR of CD36. Plasmids were transfected into beta‐TC‐6 cells using Lipofectamine 2000. After 48 h of culture, the cells were lysed. The cell lysates were prepared, and luciferase activities were measured using a Luminometer (Promega).

### 2.14. Bioinformatic Analysis

To identify potential miRNAs targeting CD36, we employed four databases: Starbase (http://starbase.sysu.edu.cn/), TargetScan (https://www.targetscan.org/), MicroT‐CDS (http://diana.imis.athena-innovation.gr/DianaTools/index.php), and mirDIP (https://ophid.utoronto.ca/mirDIP/). The miRNAs identified by all four databases were then compared with downregulated miRNAs extracted from datasets GSE168327, GSE189002, and GSE196797, obtained from the GEO database.

### 2.15. Insulin Secretion Assay

The beta‐TC‐6 cells that have undergone different treatments were equilibrated in a buffer containing 2% BSA and 2.5 mM glucose for 2 h. Then, the buffer was replaced, and the cells were incubated for another 1 h. Subsequently, the cells were incubated with KRBH buffer containing 2.5 mM glucose, 30 mM KCl, and 100 μM tolbutamide for 1 h, and then treated with KRBH buffer containing 28.0 mM glucose, 30 mM KCl, and 100 μM tolbutamide for another 1 h, which is to subject the cells to low/high sugar stimulation. Afterwards, the cell supernatants were collected, respectively. Insulin secretion levels were measured using an insulin detection instrument. Glucose‐stimulated insulin secretion (GSIS) index = Insulin secretion levels under high‐glucose stimulation/Insulin secretion levels under low‐glucose stimulation.

### 2.16. Statistical Analysis

Data were analyzed using GraphPad Prism (v8.0, USA). Unpaired two‐tailed Student’s *t*‐tests and one‐way ANOVA were used to assess significance between two or more datasets, respectively. A *p*‐value <0.05 was considered statistically significant. All experiments were performed with independent biological triplicates; data are presented as the mean ± standard deviation (SD), and technical triplicates were used for CCK‐8, qPCR, and luciferase assays.

## 3. Results

### 3.1. Characterization of BMSCs and BMSC‐EVs

To obtain BMSC‐EVs for subsequent experiments, we first characterized the multipotency of the BMSCs (Supporting Information 2: Figure S1A,B). Next, we purified EVs from the conditioned medium of these BMSCs and assessed their features. NTA and TEM results revealed that the majority of these EVs were sized from 30 to 140 nm (Figure 1A,B). Additionally, Western blot analysis showed that these EVs exhibited an enriched expression of the EV markers CD9, CD81, and CD63 (Figure 1C). Together, these results confirm the characteristics of both BMSCs and BMSC‐derived EVs.

Figure 1BMSCs and their EVs have a protective effect on hypoxic beta‐TC‐6 cells. (A) NTA results showing the size distribution of the purified BMSC‐EVs. (B) Representative TEM image depicting the morphology of the purified BMSC‐EVs. (C) Western blot data illustrating the enriched expression of EV markers CD9, CD81, and CD63 within the purified BMSC‐EVs compared to BMSCs. (D) Fluorescent microscope images showing the distribution of CFSE‐labeled BMSC‐EVs in cells. (E) CCK‐8 assay results showing the viability of cells. (F) Colony formation assay data depicting the colony formation status by cells. (G) Statistical analysis of colony formation assay data in (F). (H) Annexin V/PI staining outcomes illustrating the apoptosis of cells. (I) Quantification of the outcomes present in (H).

**Figure:**
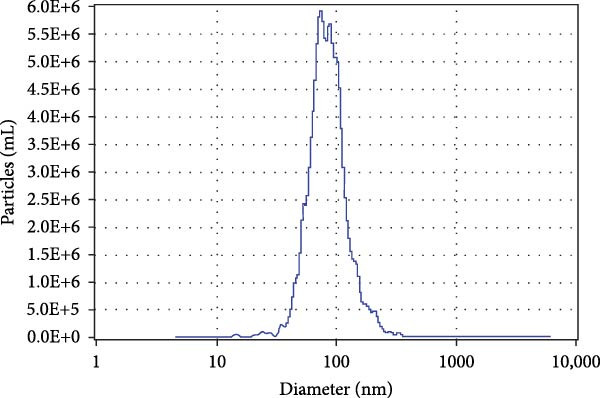
(A)

**Figure:**
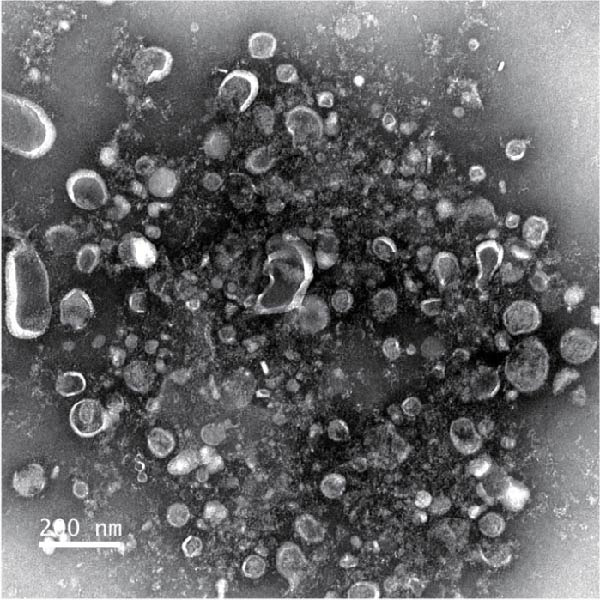
(B)

**Figure:**
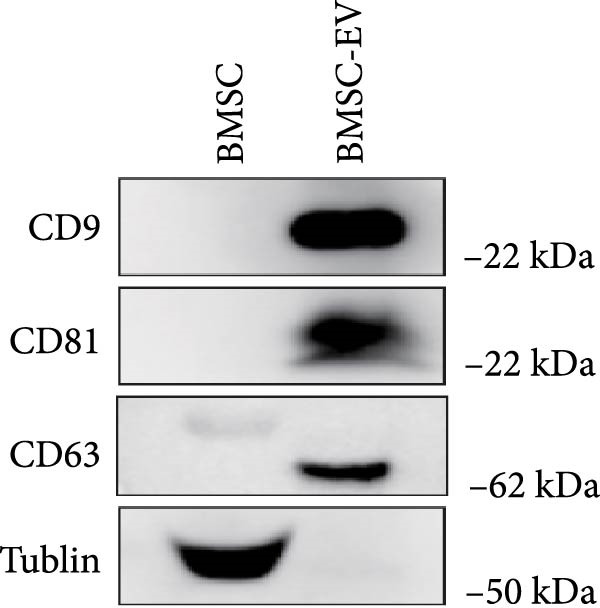
(C)

**Figure:**
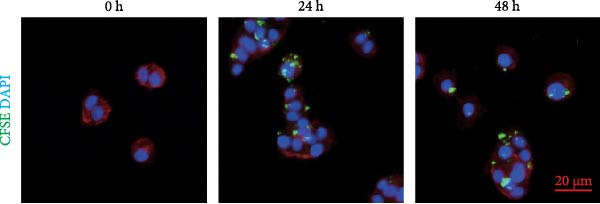
(D)

**Figure:**
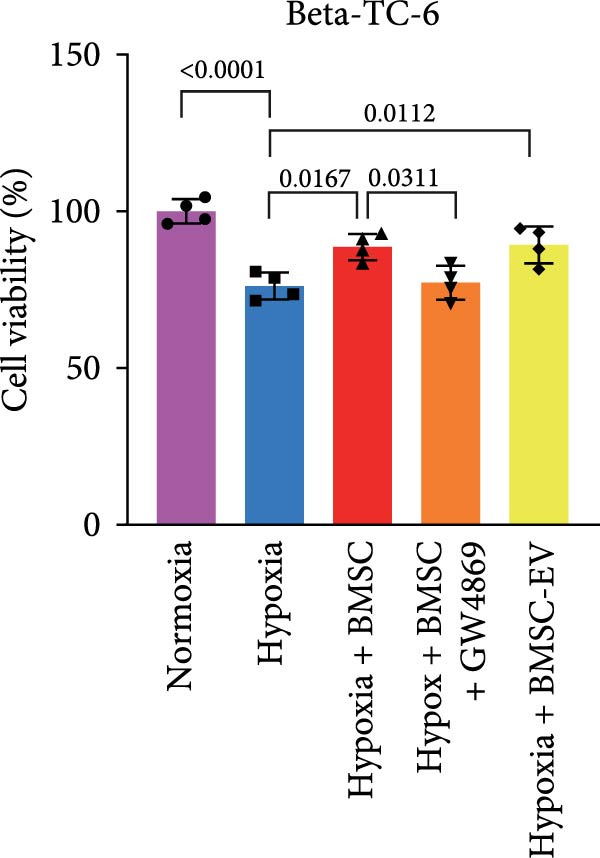
(E)

**Figure:**
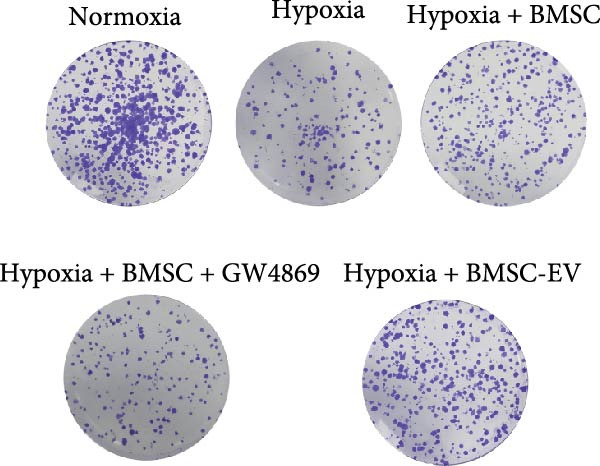
(F)

**Figure:**
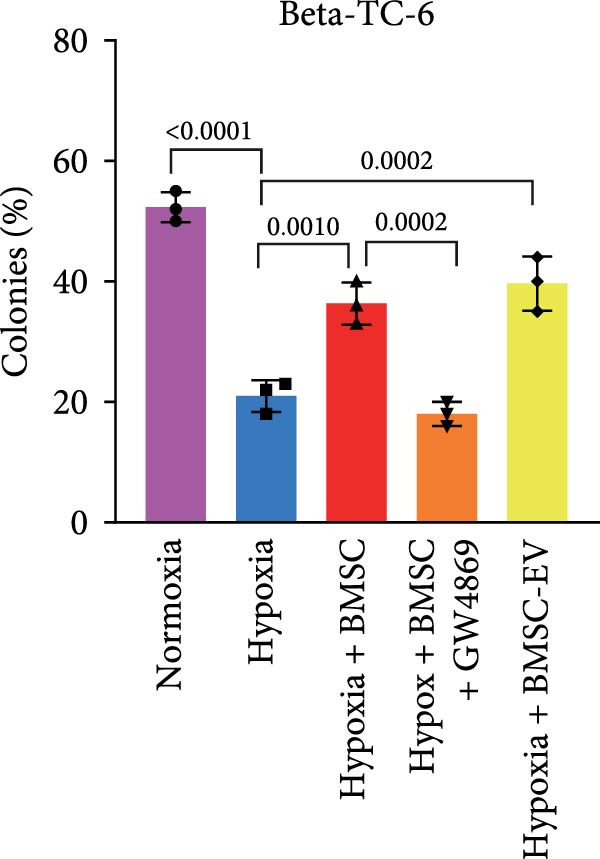
(G)

**Figure:**
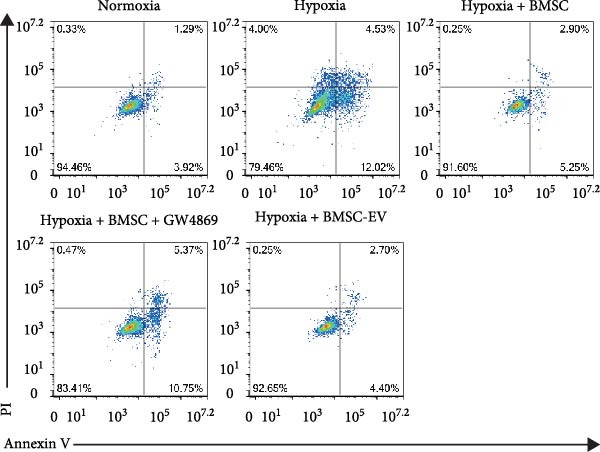
(H)

**Figure:**
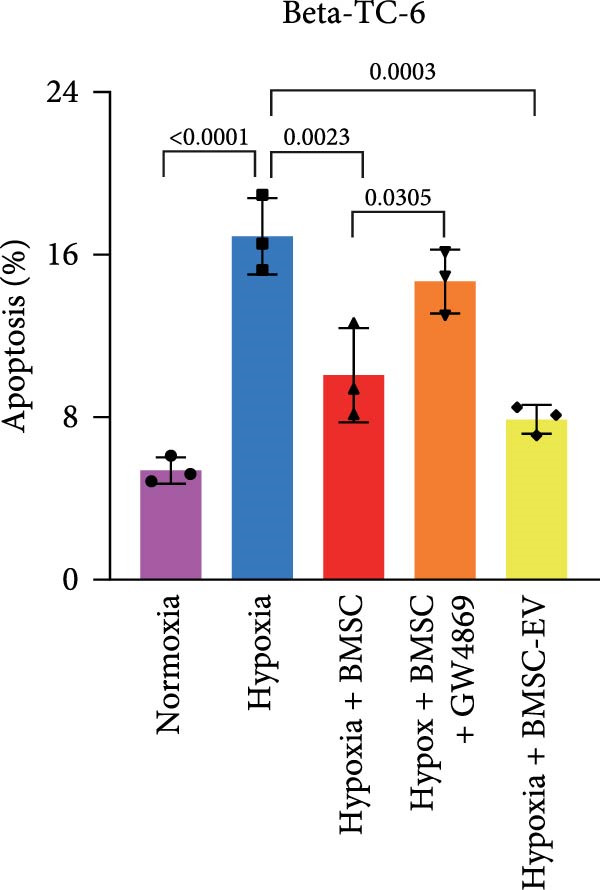
(I)

### 3.2. Influence of BMSCs and BMSC‐EVs on Hypoxia‐Challenged Beta‐TC‐6 Cells

Our subsequent investigation focused on the impact of BMSCs and BMSC‐EVs on the viability and apoptosis of pancreatic beta‐cells under hypoxic conditions, utilizing the beta‐TC‐6 cell line as a model system [27]. These cells were cultured in low‐glucose DMEM at 37°C with 2% O_2_ for 24 h. Despite maintaining a similar morphology to their normoxic counterparts, hypoxic beta‐TC‐6 cells exhibited a pronounced decline in viability and a concurrent surge in apoptotic indices, as evidenced in Supporting Information 2: Figure S2A,B. To determine whether BMSC‐EVs could be absorbed by beta‐TC‐6 cells, we treated the cells with CFSE‐labeled BMSC‐EVs for 24 or 48 h. Fluorescent signals were detected in these cells at both time points, confirming the uptake of BMSC‐EVs (Figure 1D).

For the coculture experiments, BMSCs were introduced to hypoxic beta‐TC‐6 cells within Transwell chambers at an equal ratio. The CCK‐8 assay outcomes demonstrated a significant enhancement in the viability of hypoxic beta‐TC‐6 cells upon coculture with BMSCs. Interestingly, the presence of GW4869, an inhibitor known for its ability to impede EV release [28], effectively abrogated the viability‐enhancing effect of BMSCs. In a contrasting observation, BMSC‐EVs alone were found to be equally efficacious in promoting the viability of hypoxic beta‐TC‐6 cells (Figure 1E). In alignment with these findings, the compromised colony formation capacity of hypoxic beta‐TC‐6 cells was partially revitalized by BMSC coculture, an effect that was negated by the inclusion of GW4869, as detailed in Figure 1F,G. Furthermore, Annexin V/PI staining, Western blot, and TUNEL assay results revealed that both BMSCs and BMSC‐EVs were instrumental in significantly attenuating hypoxia‐induced apoptosis. It is noteworthy that the antiapoptotic effects of BMSCs were significantly dampened by the administration of GW4869, as delineated in Figures 1H,J and 2A–D. In aggregate, these observations suggest that BMSCs may exert a protective role against hypoxia‐induced cellular injury, potentially mediated through the secretion of EVs. These results collectively underscore the therapeutic potential of BMSCs and BMSC‐EVs in mitigating cellular stress induced by hypoxic conditions, offering valuable insights into the mechanisms underlying their protective effects on pancreatic beta‐cells.

Figure 2BMSCs and BMSC‐EVs were instrumental in significantly attenuating hypoxia‐induced apoptosis. (A) Western blot data depicting the protein levels of Bax, Bcl2, Pro‐caspase 3, and Cleaved‐caspase 3 in beta‐TC‐6 cells with the indicated treatments. (B) Statistical bar graph of western blot data in (A). (C) TUNEL assay outcomes illustrating the apoptosis of beta‐TC‐6 cells with the indicated treatments. (D) Quantification of the outcomes present in (C).

**Figure:**
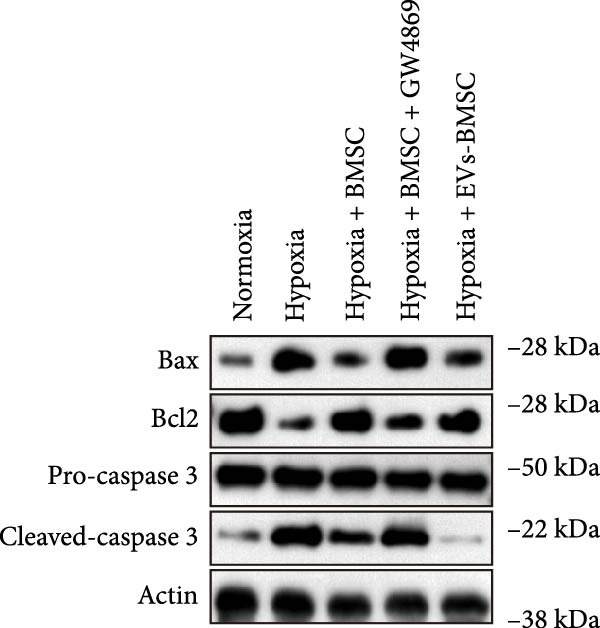
(A)

**Figure:**
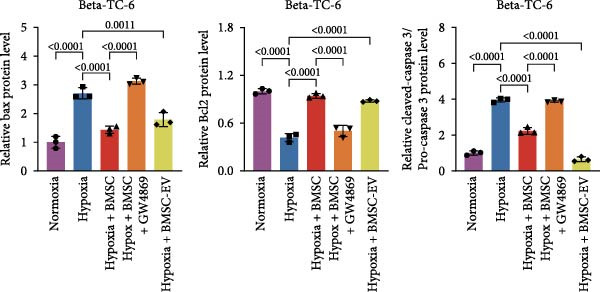
(B)

**Figure:**
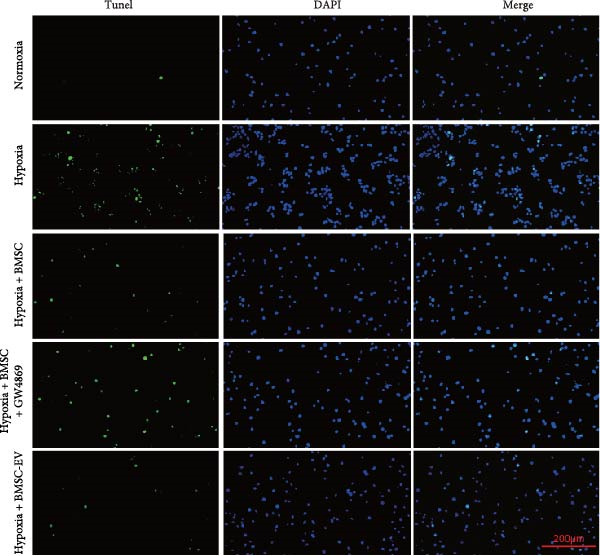
(C)

**Figure:**
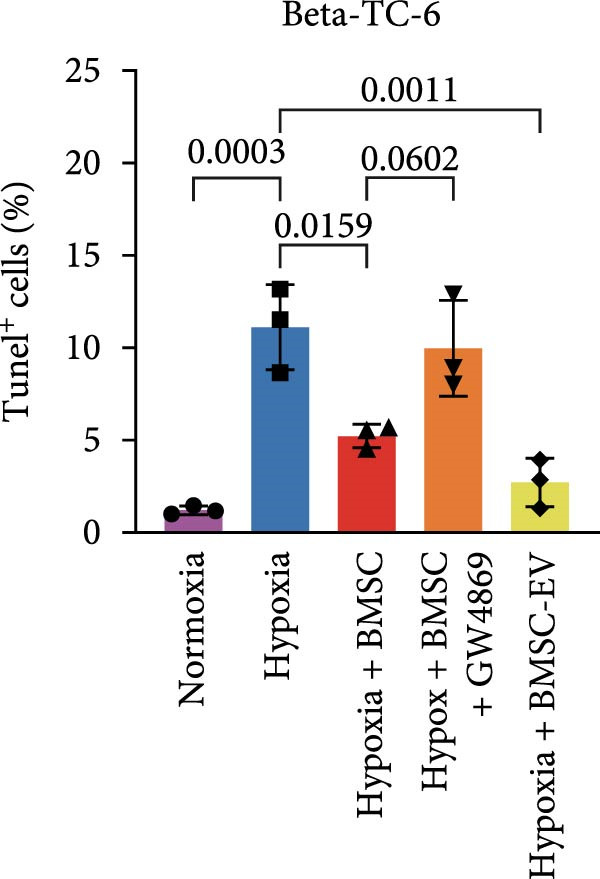
(D)

### 3.3. BMSCs and BMSC‐EVs Downregulate CD36 Expression in Hypoxia‐Treated Beta‐TC‐6 Cells

Drawing from prior research that has implicated CD36 in adverse effects on pancreatic beta‐cells [29, 30]. Furthermore, based on the chip sequencing analysis data we conducted on beta‐cells treated with normoxic, hypoxic, and hypoxic +EVs conditions (the significant difference expression threshold was set as |log2(Fold Change)| ≥2 and *p* value ≤0.01), the intersect of the differentially expressed mRNAs obtained was taken to identify the mRNAs that could counteract hypoxia‐induced damage after EVs treatment. Three mRNAs were identified, namely *Cd36*, *Gata1*, and *Krt17*. The differentially expressed gene CD36 was found to be associated with glucose intolerance, atherosclerosis, arterial hypertension, diabetes, and so on. *Gata1* was involved in hematopoiesis, *Krt17* possesses structural molecule activity and MHC class II receptor activity (data not shown). We selected the *Cd36* gene related to diabetes for the subsequent research. Thus, we sought to investigate the influence of hypoxic conditions on CD36 expression levels. Our qPCR analysis discerned a pronounced upregulation of CD36 in beta‐TC‐6 cells subjected to hypoxia. Conversely, the expression of genes integral to beta‐cell function, including PDX1, NKX6.1, insulin receptor substrate 2 (IRS‐2), and glucose transporter type 2 (GLUT2) [31–34], was significantly diminished under hypoxic stress. In parallel, the expression of uncoupling protein 2 (UCP2), a gene associated with the impairment of beta‐cell secretory function [35], was induced by hypoxia (Figure 3A).

Figure 3BMSCs and BMSC‐EVs decrease CD36 expression in hypoxic beta‐TC‐6 cells. (A) qPCR results showing the mRNA expression of CD36, PDX1, NKX6.1, IRS‐2, GLUT2, and UCP2 in cells with the indicated treatments. (B, D) Western blot data depicting the protein levels of CD36, PI3K, P‐PI3K, AKT, and P‐AKT in beta‐TC‐6 cells with the indicated treatments. (C, E) Statistical bar graph of Western blot data in (B, D).

**Figure:**
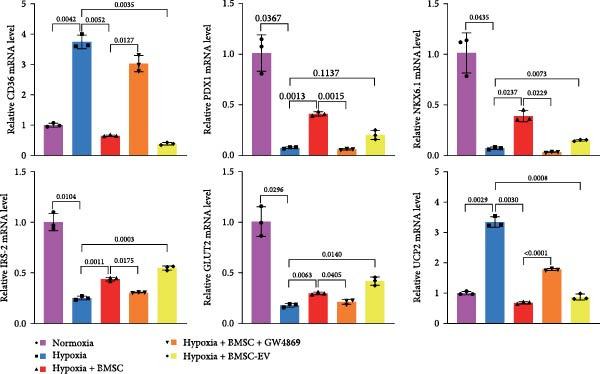
(A)

**Figure:**
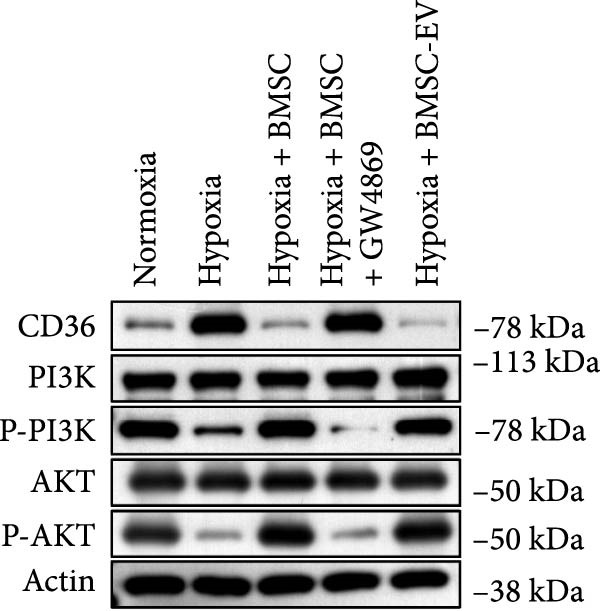
(B)

**Figure:**
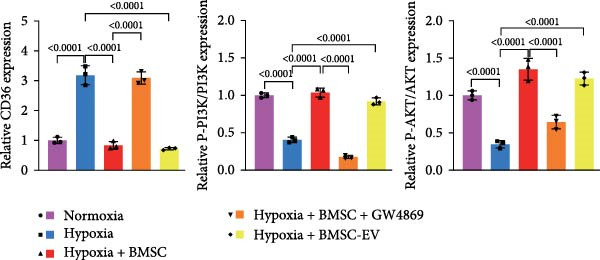
(C)

**Figure:**
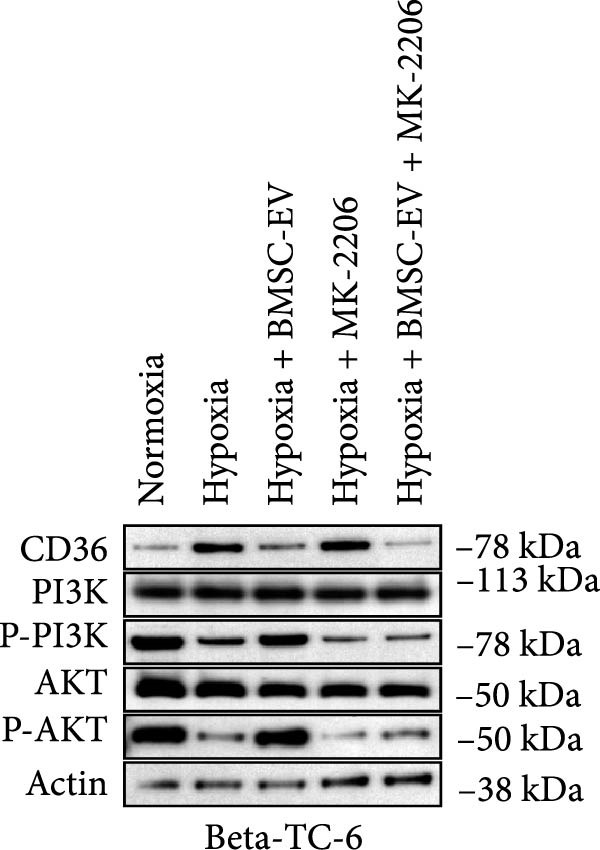
(D)

**Figure:**
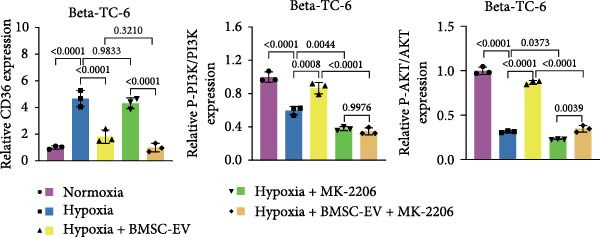
(E)

Notably, the inhibitory effect of hypoxia on gene expression was significantly alleviated by the coculture of BMSCs or BMSC‐EVs. However, the protective effect of BMSCs was compromised by GW4869, an inhibitor of EV release (Figure 3A). It is noteworthy that BMSC‐EVs showed a less efficacy in restoring PDX1 mRNA levels compared to BMSCs (Figure 3A). These findings were corroborated by Western blot analysis, which indicated that BMSCs or BMSC‐EVs could counteract the hypoxia‐induced upregulation of CD36. This reversal was not observed in BMSCs pretreated with GW4869. Given the established role of CD36 in the repression of the PI3K/AKT signaling pathway [30], we further examined the status of this pathway by assessing the levels of phosphorylated PI3K (P‐PI3K) and AKT (P‐AKT). Our Western blot analysis revealed that BMSCs and BMSC‐EVs could restore the hypoxia‐depleted levels of P‐PI3K and P‐AKT. However, GW4869 treatment abrogated the BMSC‐mediated activation of the PI3K/AKT pathway in cocultured hypoxic cells (Figure 3B,C). We further verified the significance of the PI3K/AKT pathway in CD36 regulation by introducing the pathway inhibitor MK‐2206. By measuring the insulin secretion levels of each group of cells under low/high glucose stimulation and calculating the GSIS index, the functional changes of the beta‐cells were reflected. The Western blot results showed that MK‐2206 did not affect the effects of hypoxia or BMSC‐EVs on the expression of CD36. Under low‐glucose stimulation, there was no significant difference in insulin secretion levels among the various groups of cells. However, MK‐2206 further inhibited the PI3K/AKT pathway and insulin secretion (under high glucose stimulation) in hypoxic cells, and MK‐2206 also significantly suppressed the activation effects of BMSC‐EVs on the PI3K/AKT pathway and insulin secretion (Figure 3D,E, Supporting Information 2: Figure S3A,B). Collectively, these results suggest that BMSCs and BMSC‐EVs may confer protection upon hypoxia‐stressed beta‐TC‐6 cells, in part, by downregulating CD36 expression. The PI3K/AKT pathway is indispensable in this regulatory process. These findings suggest that BMSCs and BMSC‐EVs may exert their protective effects on hypoxia‐induced beta‐TC‐6 cells by downregulating CD36 expression.

### 3.4. Screening of CD36‐Targeting miRNAs That Improve the Viability of Beta‐TC‐6 Cells

To elucidate the mechanism by which BMSCs and BMSC‐EVs regulate CD36 expression, we directed our focus toward miRNAs, given their pivotal roles in the regulatory functions of EVs across diverse biological contexts [36, 37]. Through a comprehensive search for miRNAs with the potential to target CD36 across four reputable databases, we identified a cohort of nine candidate miRNAs. These were further cross‐referenced with miRNAs known to be downregulated in patients with T2D. This analysis yielded three promising candidates: miR‐155‐5p, miR‐539‐3p, and miR‐485‐3p (Figure 4A,B). Subsequent qPCR analysis to assess their expression levels under normoxic and hypoxic conditions revealed that miR‐155‐5p and miR‐485‐3p were upregulated, while miR‐539‐3p was downregulated in hypoxia‐treated beta‐TC‐6 cells (Figure 4C).

Figure 4Screening of miRNAs targeting CD36 that improve the activity of beta‐TC‐6 cells. (A, B) Venn diagrams showing the miRNA screening results utilizing the indicated databases or datasets. (C) qPCR results illustrating the relative expression of miR‐155‐5p, miR‐539‐3p, and miR‐485‐3p in beta‐TC‐6 cells. (D) qPCR data indicating the expression of CD36. (E) CCK‐8 assay outcomes depicting the viability of cells. (F) Luciferase activity data indicating the interaction between miR‐539‐3p and WT or mutant 3’UTR of CD36.

**Figure:**
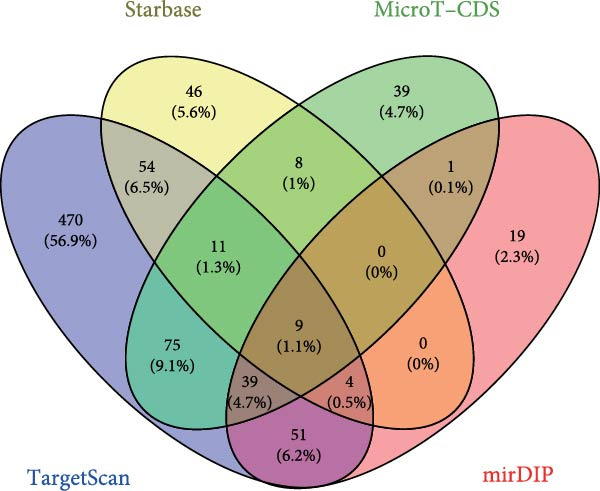
(A)

**Figure:**
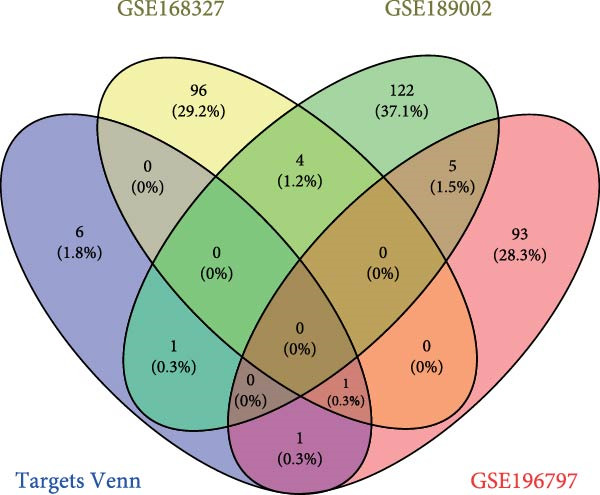
(B)

**Figure:**
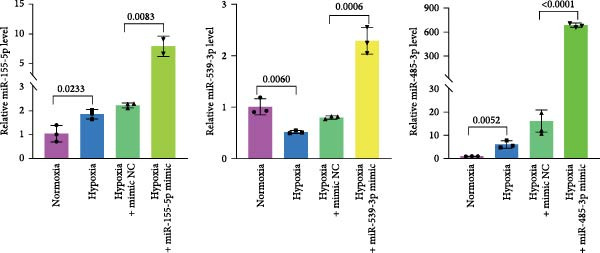
(C)

**Figure:**
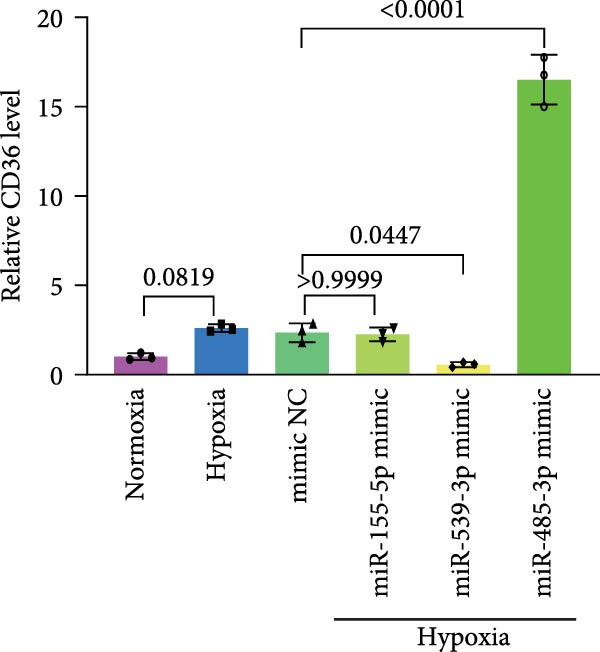
(D)

**Figure:**
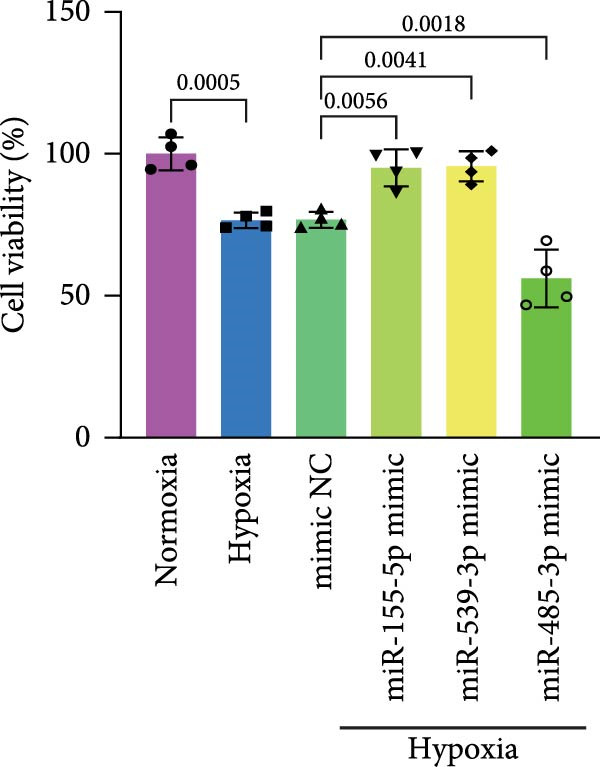
(E)

**Figure:**
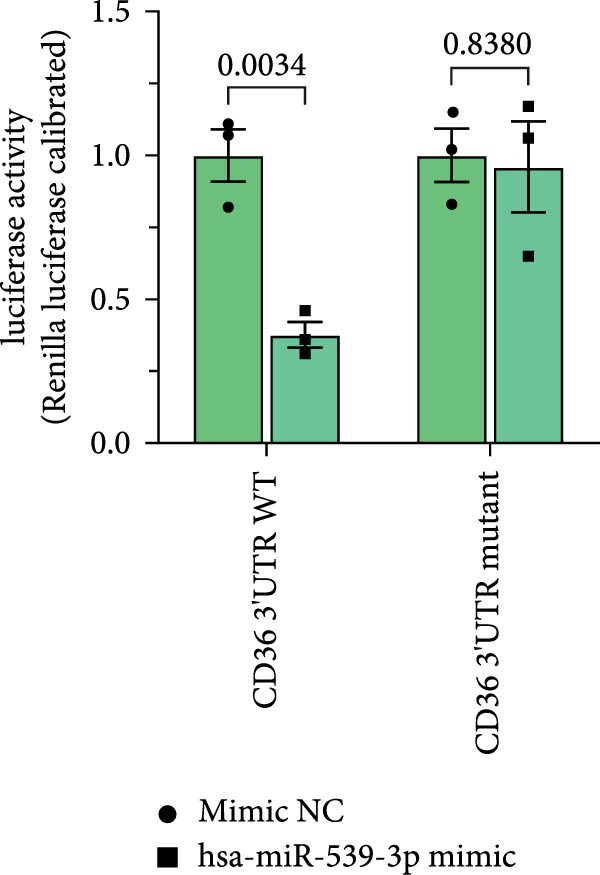
(F)

We then investigated the impact of these miRNA mimics on CD36 expression in hypoxia‐induced beta‐TC‐6 cells. qPCR confirmed the successful overexpression of the miRNAs (Figure 4C) and further disclosed that miR‐155‐5p had a negligible effect on CD36 expression. In contrast, miR‐539‐3p mimic robustly suppressed CD36 levels, and miR‐485‐3p significantly induced CD36 expression in hypoxic beta‐TC‐6 cells (Figure 4D). Interestingly, miR‐155‐5p and miR‐539‐3p mimics were able to rescue the hypoxia‐impaired cell viability, whereas miR‐485‐3p mimic exacerbated the impairment of these cells (Figure 4E). Additionally, we constructed luciferase reporters containing the WT and mutant 3’UTR of CD36. Luciferase activity assays confirmed that miR‐539‐3p binds to and represses the WT CD36 3’UTR, an interaction that was absent with the mutant 3’UTR (Figure 4F). These findings suggest that hypoxia may upregulate CD36 expression by downregulating miR‐539‐3p.

### 3.5. miR‐539‐3p Inhibitor Reverses the Protective Effects of BMSCs and BMSC‐EVs on Hypoxia‐Treated Beta‐TC‐6 Cells

We also examined the expression of miR‐539‐3p in BMSCs and BMSC‐EVs, and found that it was highly expressed in BMSC‐EVs (Supporting Information 2: Figure S4A). To confirm the critical role of miR‐539‐3p in mediating the protective effects of BMSCs and BMSC‐EVs on hypoxic beta‐TC‐6 cells, we utilized a miR‐539‐3p inhibitor during coculture. Firstly, we downregulated the expression of miR‐539‐3p in BMSCs (Supporting Information 2: Figure S4B). Our results indicated that the miR‐539‐3p inhibitor nullified the viability‐enhancing effects of BMSCs or BMSC‐EVs (Figure 5A). Moreover, the inhibitor hindered the ability of BMSCs or BMSC‐EVs to restore the impaired colony formation capacity of hypoxic beta‐TC‐6 cells, as demonstrated by colony formation assays (Figure 5B,C). Furthermore, Annexin V/PI staining revealed that the presence of the miR‐539‐3p inhibitor negated the anti‐apoptotic effects of BMSCs or BMSC‐EVs in hypoxic beta‐TC‐6 cells (Figure 5D,E). These results highlight the indispensable role of miR‐539‐3p in the protective mechanisms of BMSCs and BMSC‐EVs.

Figure 5miR‐539‐3p inhibition mitigates the protective effect of BMSCs and BMSC‐EVs on hypoxic beta‐TC‐6 cells. (A) CCK‐8 assay data showing the viability of cells. (B) Colony formation assay results depicting the colony formation status. (C) Statistical analysis of colony formation assay data in (B). (D) Annexin V/PI staining outcomes illustrating the apoptosis of beta‐TC‐6 cells. (E) Quantification of the outcomes present in (D).

**Figure:**
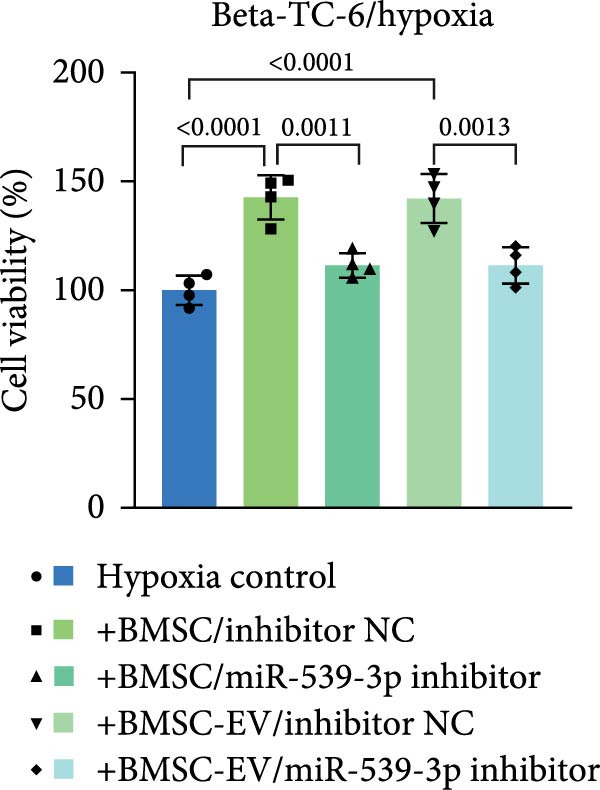
(A)

**Figure:**
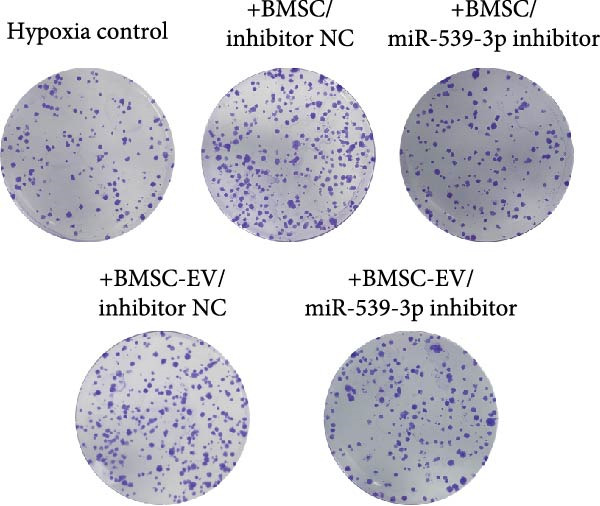
(B)

**Figure:**
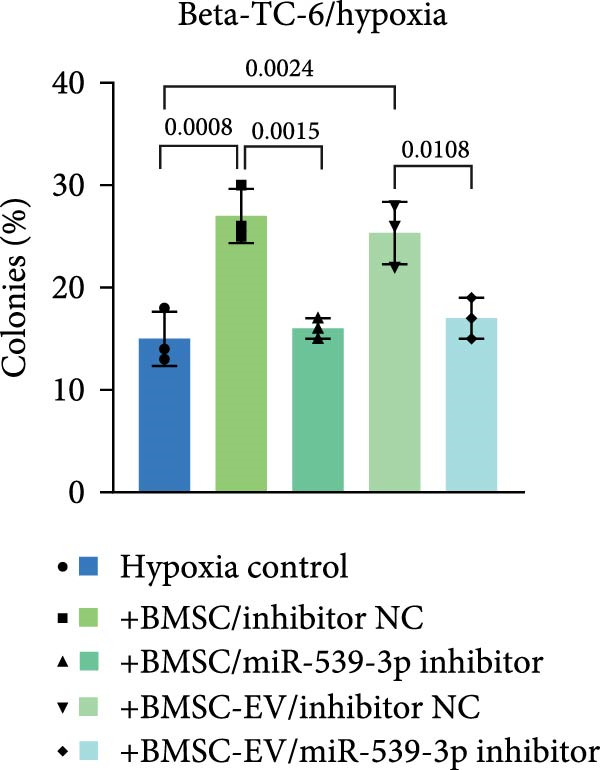
(C)

**Figure:**
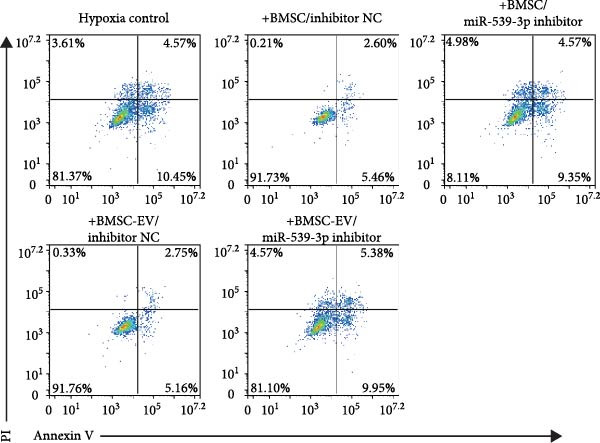
(D)

**Figure:**
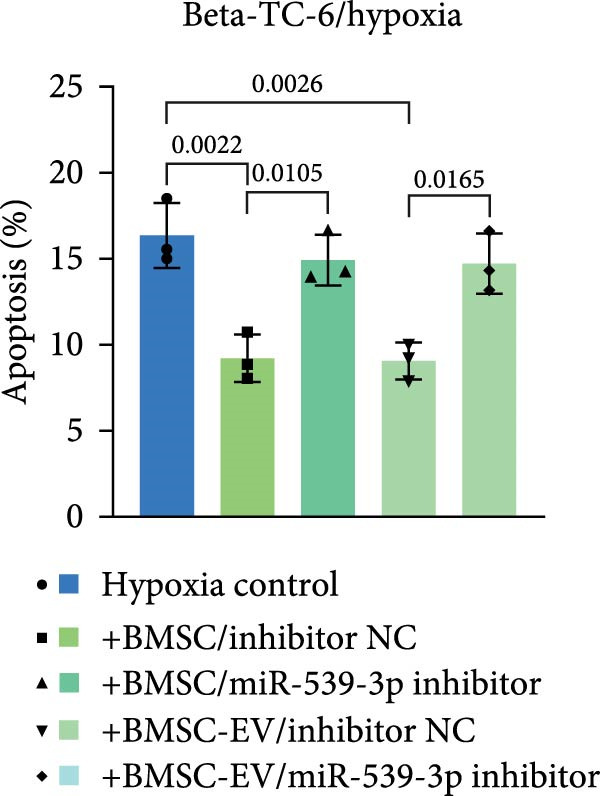
(E)

### 3.6. miR‐539‐3p Inhibition Attenuates the Ability of BMSCs and BMSC‐EVs to Suppress CD36 Expression

To further evaluate the effect of miR‐539‐3p inhibition on CD36 expression and the expression of other genes critical to beta‐TC‐6 cell functionality, we performed qPCR. Our findings indicated that coculture with BMSCs or BMSC‐EVs led to an upregulation of miR‐539‐3p levels in hypoxia‐induced beta‐TC‐6 cells (Figure 6A). Under hypoxic conditions, the expression of CD36 and UCP2 was induced. However, treatment with BMSCs or BMSC‐EVs downregulated CD36 and UCP2 and upregulated genes associated with beta cell functionality, including PDX1, NKX6.1, IRS‐2, and GLUT2. The presence of a miR‐539‐3p inhibitor attenuated these effects (Figure 6B). Western blot analysis further substantiated these findings, showing that BMSCs and BMSC‐EVs inhibited the upregulation of CD36 protein levels in hypoxia‐induced beta‐TC‐6 cells. Moreover, miR‐539‐3p inhibition disrupted the ability of BMSCs and BMSC‐EVs to suppress CD36 levels and to enhance the levels of P‐PI3K and P‐AKT in hypoxic beta‐TC‐6 cells (Figure 6C,D). These data suggest that miR‐539‐3p likely functions in hypoxia‐induced beta‐TC‐6 cells by targeting CD36 expression.

Figure 6Excessive miR‐539‐3p inhibitor mitigates the inhibitory effect of BMSC and BMSC‐EVs on CD36 expression in hypoxic beta‐TC‐6 cells. (A) qPCR data showing the relative expression of miR‐539‐3p. (B) qPCR results indicating the mRNA levels of CD36, PDX1, NKX6.1, IRS‐2, GLUT2, and UCP2 in cells. (C) Western blot data depicting the protein levels of CD36, PI3K, P‐PI3K, AKT, and P‐AKT. (D) Statistical bar graph of Western blot data shown in (C).

**Figure:**
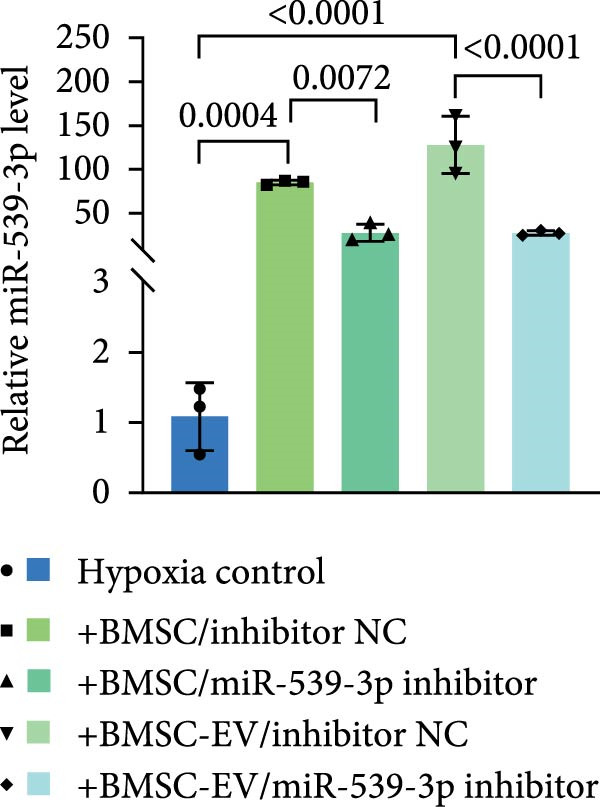
(A)

**Figure:**
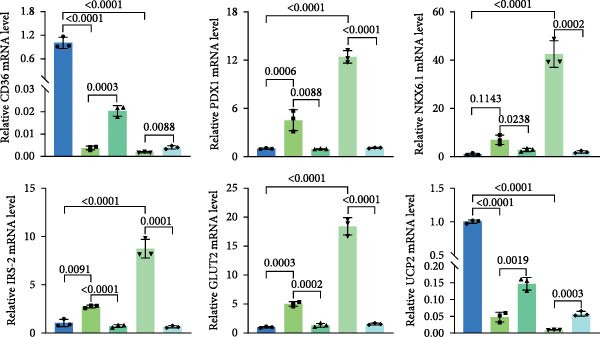
(B)

**Figure:**
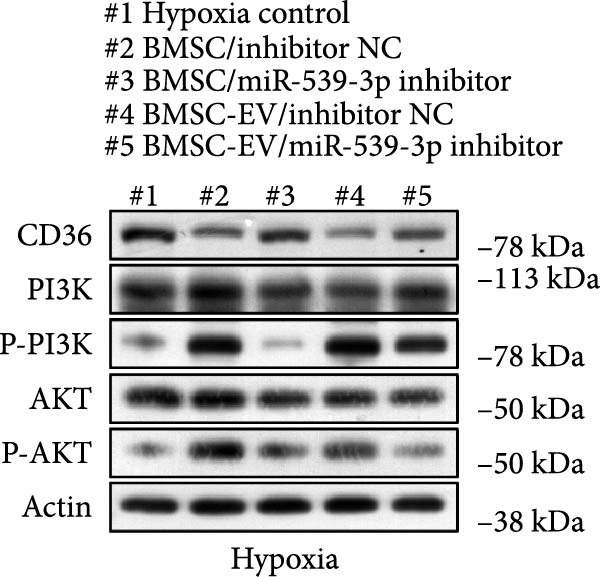
(C)

**Figure:**
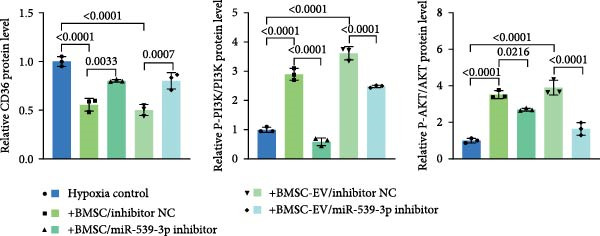
(D)

### 3.7. CD36 Knockdown in Hypoxic Beta‐TC‐6 Cells Counterbalances the Impact of miR‐539‐3p Inhibition on BMSCs and Their BMSC‐EVs Effects

To further validate the miR‐539‐3p‐CD36 axis in hypoxia‐induced beta‐TC‐6 cells, we performed CD36 knockdown. qPCR confirmed the efficient depletion of CD36 by three shRNAs, with shCD36‐1 being selected for further experiments (Supporting Information 2: Figure S5). CCK‐8 assays demonstrated that CD36 knockdown enhanced the viability of hypoxic beta‐TC‐6 cells (Figure 7A). Consistently, colony formation assays and Annexin V/PI staining data indicated that CD36 knockdown mitigated the effects of miR‐539‐3p inhibition on colony formation ability and apoptosis in hypoxic cells (Figure 7B–E). At the molecular level, CD36 knockdown in hypoxia‐induced beta‐TC‐6 cells resulted in elevated expression levels of PDX1, NKX6.1, IRS‐2, and GLUT2, while concurrently reducing UCP2 expression (Figure 8A). Additionally, CD36 knockdown significantly increased the levels of P‐PI3K and P‐AKT, as demonstrated by Western blot analysis (Figure 8B,C). This result confirmed that CD36 regulates the activation of its downstream PI3K/AKT pathway.

Figure 7Deficiency of CD36 attenuates the impairment of hypoxia‐induced beta‐TC‐6 cells. (A) CCK‐8 assay data showing the cell viability. (B) Colony formation assay results depicting the colony formation status. (C) Statistical analysis of colony formation assay data in (B). (D) Annexin V/PI staining outcomes illustrating the apoptosis. (E) Quantification of the outcomes present in (D).

**Figure:**
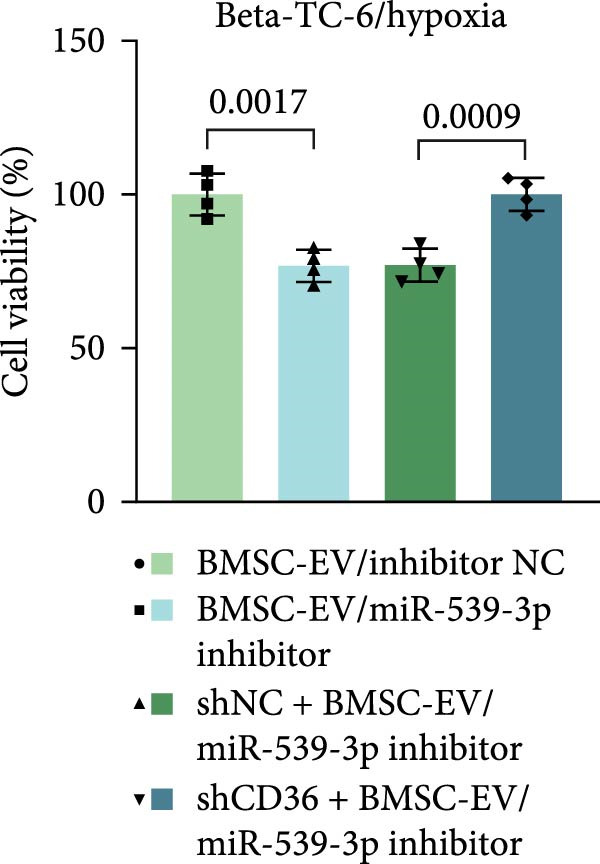
(A)

**Figure:**
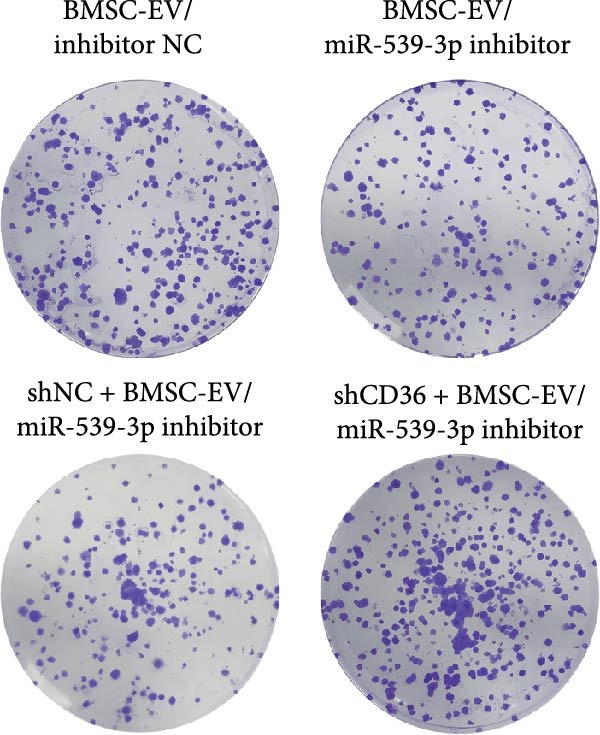
(B)

**Figure:**
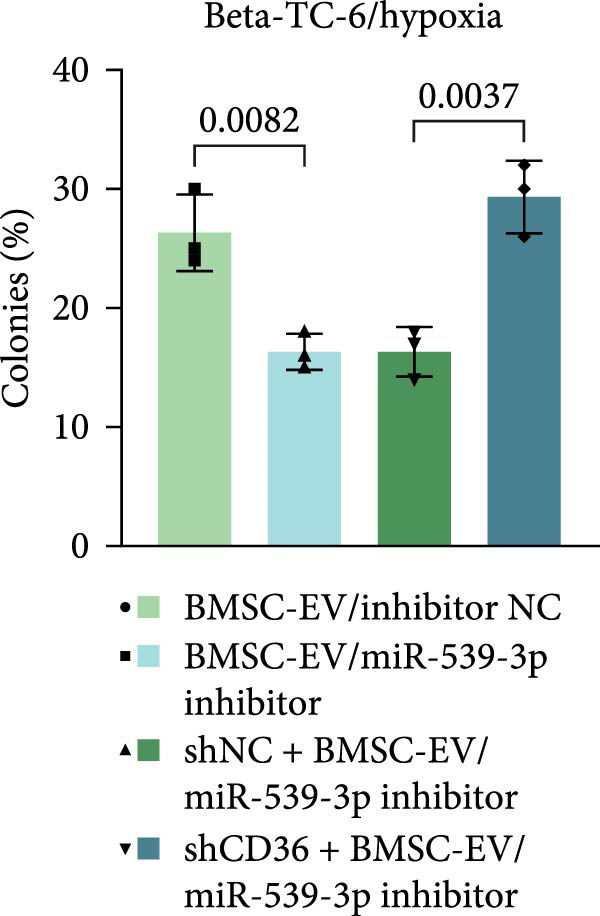
(C)

**Figure:**
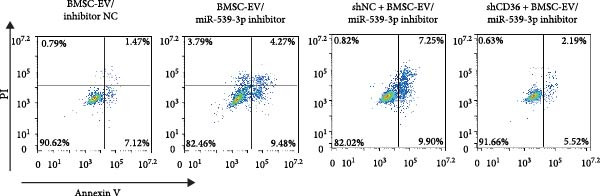
(D)

**Figure:**
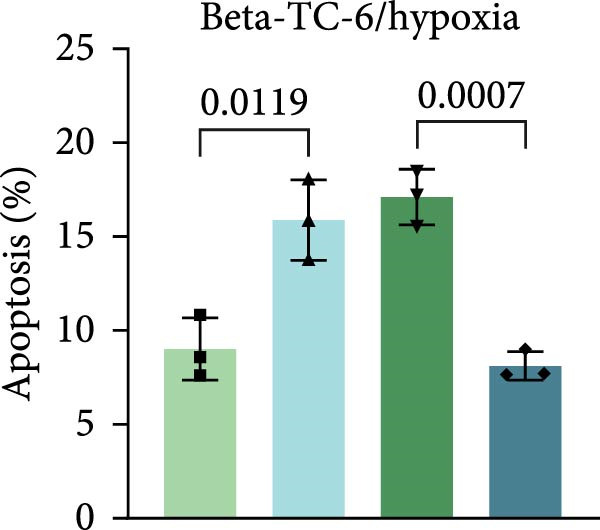
(E)

Figure 8Knockdown of CD36 balances the impact of miR‐539‐3p inhibitor on the effect of BMSCs and their EVs in hypoxic beta‐TC‐6 cells. (A) qPCR results indicating the mRNA levels of CD36, PDX1, NKX6.1, IRS‐2, GLUT2, and UCP2. (B) Western blot data depicting the protein levels of CD36, PI3K, P‐PI3K, AKT, and P‐AKT. (C) Statistical bar graph of Western blot data shown in (B).

**Figure:**
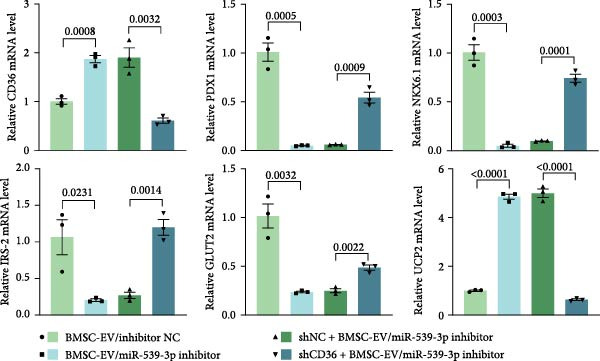
(A)

**Figure:**
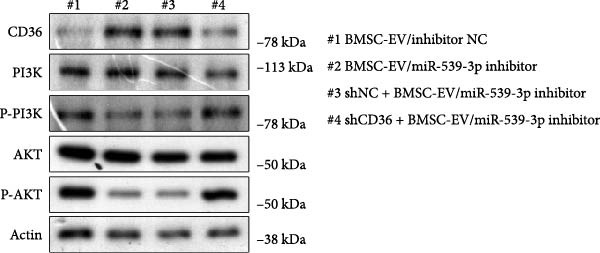
(B)

**Figure:**
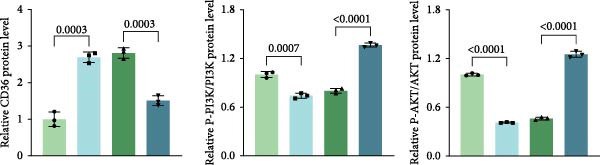
(C)

We also measured the insulin secretion levels (under low/high‐glucose stimulation) and GSIS index in beta‐TC‐6 cells under different treatment conditions, and the results are shown in Supporting Information 2: Figure S3C,D. Under high‐glucose stimulation, hypoxic beta‐TC‐6 cells exhibited a significant decrease in insulin secretion levels and GSIS index. BMSC‐EVs can alleviate the damage caused by hypoxic conditions, but this effect is inhibited by the miR‐539‐3p inhibitor. Similarly, CD36 knockdown mitigated the impacts of miR‐539‐3p inhibition on the insulin secretion levels of hypoxic cells. These findings further corroborate the notion that miR‐539‐3p confers protection to beta‐TC‐6 cells against hypoxia‐induced injury by targeting CD36.

## 4. Discussion

The therapeutic potential of BMSCs in addressing pancreatic beta‐cell dysfunction has garnered considerable attention. Our study consistently demonstrates that BMSCs extend their protective influence to hypoxic beta‐TC‐6 cells, a finding that is particularly significant. Although BMSCs possess the capability to differentiate into beta‐cells, such events occur infrequently in vivo, suggesting that their regenerative and functional stimulatory effects on beta‐cells are predominantly mediated through paracrine actions [38]. This hypothesis is substantiated by our observations that the EV secretion inhibitor GW4869 significantly diminishes the protective effects of BMSCs on hypoxic beta‐TC‐6 cells, underscoring the indispensable role of BMSC‐EVs in the cellular crosstalk.

miRNAs are one of the major functional biomolecules enclosed in MSC‐derived EVs, and their regulatory function in diabetes has been extensively explored [39]. However, their specific impact on hypoxic beta‐cells remains to be fully elucidated. A recent study reports that miR‐101a‐3p encapsulated in BMSC‐EVs can alleviate hypoxia‐reperfusion injury of cardiomyocytes [40], indicating the vital role of miRNAs in mediating the protective effect of BMSC‐EVs against hypoxia. In line with this, our data showing that miR‐539‐3p controls the level of CD36 in hypoxic beta‐TC‐6 cells, thereby alleviating the injury triggered by hypoxia in beta‐TC‐6 cells, further underscores the importance of miRNAs in mediating the function of EVs.

Hypoxia is known to significantly deplete miR‐539‐3p levels in beta‐TC‐6 cells, a deficit that can be remedied by BMSCs or BMSC‐EVs. The restoration of miR‐539‐3p in these cells could be attributed to the miR‐539‐3p enclosed within BMSC‐EVs, which are internalized by recipient beta‐TC‐6 cells. Alternatively, BMSCs or their EVs might modulate the hypoxia‐induced downregulation of miR‐539‐3p or enhance its transcriptional machinery. Further research is necessary to explore these possibilities.

CD36, recognized as a repressor of beta‐cell function and survival, may contribute to hypoxia‐induced injury through its upregulated expression. Interestingly, the CD36 protein level in beta cells is negatively regulated by insulin, which facilitates its ubiquitination and degradation [41]. The reduced insulin secretion by dysfunctional beta cells in hypoxia may thus induced upregulation of CD36. Furthermore, as a fatty acid transporter/scavenger receptor, the upregulation of CD36 in beta‐cells may lead to lipotoxicity, oxidative stress, or metabolic dysfunction. Studies have shown that overexpression of CD36 increases the uptake of oxLDL, inducing a shift in mitochondrial metabolism from oxidative phosphorylation to superoxide production. CD36 can also mediate oxLDL uptake through JNK activation and downstream pro‐apoptotic signaling, inducing ER stress, leading to dysfunction and apoptosis [29, 42].

Ceramide activates the Src‐Vav2 signaling pathway, leading to the activation of NADPH oxidase and an increase in ROS production, thereby promoting redoxosome formation. CD36 is a key mediator in this pathway and is activated by ceramide, amplifying oxidative stress, mitochondrial dysfunction, and beta‐cell apoptosis [43]. Existing evidence shows that inducing the expression of CD36 under hyperglycemic conditions leads to an increase in free fatty acid uptake and ROS production, resulting in mitochondrial dysfunction and beta‐cell failure, thereby impairing insulin secretion and promoting beta‐cell apoptosis [29, 44]. Inhibiting CD36 can restore insulin secretion and reduce oxidative stress [45]; this is consistent with our research findings. Therefore, targeting the upregulated CD36 under hypoxic conditions to regulate fatty acid uptake and inhibit oxidative stress may represent a potential therapeutic strategy for combating beta‐cell dysfunction. In this study, the increased CD36 levels could also be ascribed to the influence of miRNAs, such as miR‐485‐3p, which potently induce CD36 expression. However, the underlying mechanisms warrant further investigation.

The functional repertoire of miR‐539‐3p has been explored in diverse cellular contexts. For instance, miR‐539‐3p acts as a tumor suppressor by inhibiting CDK14 in colon cancer cells [29], while it promotes ovarian cancer by targeting SPARCL1 [42]. This duality underscores the context‐dependent nature of miR‐539‐3p function, which is largely dictated by its target mRNAs. It would be insightful to investigate whether miR‐539‐3p regulates the expression of other genes in hypoxic beta cells.

It is noteworthy that our conclusion is exclusively based on the impact of BMSCs and BMSC‐EVs on the viability, colony formation ability, and survival of hypoxic beta‐TC‐6 cells. The potential of BMSCs or BMSC‐EVs to enhance the secretory function of these cells remains to be determined. Additionally, the in vivo applicability of these mechanisms in protecting hypoxic beta cells require further exploration. Besides, the protective effects exerted by BMSCs and BMSC‐EVs are slightly different in certain aspects. For instance, BMSC‐EVs are slightly less effective than whole BMSCs in restoring the Pdx1 mRNA level in hypoxic cells. This might be attributed to the fact that BMSCs secrete additional soluble factors (such as cytokines, growth factors, etc.), which interact synergistically with BMSC‐EVs to optimally promote the functional recovery of beta‐cells, the possible mechanisms of the synergistic effect may include: the soluble factors activate the signaling pathways within the target cells, making them more responsive to the signals from EVs. Additionally, the soluble factors and EVs act on different targets of the same pathological process and work together through complementary mechanisms. The related mechanisms involved in beta‐TC‐6 cells are worthy of further exploration.

## 5. Conclusion

In conclusion, our study elucidates a novel mechanism by which BMSCs and their EVs ameliorate hypoxia‐induced injury in beta‐TC‐6 cells through the regulation of the miR‐539‐3p‐CD36 axis. This discovery not only enhances our understanding of BMSC‐mediated beta cell protection but also suggests that targeting this axis, through the overexpression of miR‐539‐3p or the knockdown of CD36, may unlock new therapeutic avenues for BMSCs and their EVs in treating beta cell dysfunction in diabetic patients. This targeted approach could potentially enhance the therapeutic efficacy of BMSCs and their EVs, offering a promising strategy for diabetes management.

**Nomenclature**
BMSCs: Bone marrow mesenchymal stromal cells
CFSE: Carboxyfluorescein succinimidyl ester
ER: ER: Endoplasmic reticulum
EVs: Extracellular vesicles
GLUT2: Glucose transporter type 2
IRS‐2: Insulin receptor substrate 2
MOI: Multiplicity of infection
MSCs: Mesenchymal stromal cells
NKX6.1: NK homeobox 1
NC: Negative control
PDX‐1: Pancreatic duodenal homeobox factor‐1
ROS: Reactive oxygen species
RT: Room temperature
T1DM: Type 1 diabetes
T2DM: Type 2 diabetes
UCP2: Uncoupling protein 2.

## Ethics Statement

The project was approved by the Animal Care and Use Committee of Hainan Medical University (HYLL‐2022‐383) on October 8, 2022, and by the Institutional Animal Care and Use Committee of Fujian Medical University (IACUC FJMU2024‐Y‐2095) on August 18, 2024.

## Disclosure

All authors have read and approved the final manuscript.

## Conflicts of Interest

The authors declare no conflicts of interest.

## Author Contributions

Conceptualization: Jin Chen and Lingfeng Zhu. Funding acquisition: Jin Chen and Na Lin. Data curation: Na Lin, Yaoyao Liang, and Zhuoyu Li. Investigation: Na Lin, Yaoyao Liang, and Minying Tang. Methodology: Fei Liu, Liuyan Chen, and Yunfeng Fu. Formal analysis: Lvying Wu. Supervision: Lingfeng Zhu. Writing – original draft: Jin Chen. Writing – review and editing: Jin Chen and Lingfeng Zhu. Na Lin, Yaoyao Liang, Minying Tan, and Fei Liu contributed equally to this work and shared the first authorship.

## Funding

This work was supported by grants from the National Natural Science Foundation of China (82260161), the Natural Science Foundation of Fujian Province (2021J011266 and 2025J01278), the Natural Science Foundation of Hainan Province (822MS179), the Key Research and Development Project of Hainan Province (ZDYF2024SHFZ062), and the Science and Technology Planning project of Fuzhou (2024‐S‐092).

## Supporting Information

Additional supporting information can be found online in the Supporting Information section.

## Supporting information


**Supporting Information 1** Table S1: Primers for plasmid construction and miRNA mimic/inhibitor. Table S2: Western blot antibody information. Table S3: qPCR primers.

**Supporting Information 2** Figure S1: Characterization of BMSCs. Figure S2: Establishment of hypoxia cellular model. Figure S3: Detection of insulin secretion levels and glucose‐stimulated insulin secretion (GSIS) index in beta‐TC‐6 cells under different treatments. Figure S4: Detection of miR‐539‐3p level. Figure S5: Validation of the efficiency of shRNAs targeting CD36. Figure S6: Full‐length blots of western blotting.

## Data Availability

The data that support the findings of this study are available from the corresponding author upon reasonable request.
