# Supplementary material for: Bone Marrow Mesenchymal Stromal Cells and Their Derived Extracellular Vesicles Protect Pancreatic Beta‐TC‐6 Cells From Hypoxia‐Induced Injury via miR‐539‐3p‐Mediated Downregulation of CD36 Expression
Source: Stem Cells Int. 2026 Jan 21;2026:6616986. doi: 10.1155/sci/6616986 (PMC12824418; doi:10.1155/sci/6616986)
Supplement: Supplementary file 1 — Supporting Information 1 Table S1: Primers for plasmid construction and miRNA mimic/inhibitor. Table S2: Western blot antibody information. Table S3: qPCR primers. [file SCI-2026-6616986-s002.docx]

**Supplementary Tables**

| **Table S1. Primers for plasmid construction and miRNA mimic/inhibitor.** | |
| --- | --- |
| **Primer** | **Sequence 5’-3’** |
| pmirGLO-Cd36-3'UTR-WT-F | TAGCCTCGAGTCTAGAtaatcaaaactgcatggcag |
| pmirGLO-Cd36-3'UTR-WT-R | ATGCCTGCAGGTCGACttctcatacactcttccagc |
| pmirGLO-Cd36-3'UTR-Mut-F | TAGCCTCGAGTCTAGAtaatcaaaactgcatggcagc |
| pmirGLO-Cd36-3'UTR-Mut-F | ATGCCTGCAGGTCGACttcactcttccagcctctttaaattc |
| shCD36-1-F | CCGGggaagttgtccttgaagaaggCTCGAGccttcttcaaggacaacttccTTTTT |
| shCD36-1-R | AATTAAAAAggaagttgtccttgaagaaggCTCGAGccttcttcaaggacaacttcc |
| shCD36-2-F | CCGGgcaccactgtgtacagacagtCTCGAGactgtctgtacacagtggtgcTTTTT |
| shCD36-2-R | AATTAAAAAgcaccactgtgtacagacagtCTCGAGactgtctgtacacagtggtgc |
| shCD36-3-F | CCGGgctattgcgacatgattaatgCTCGAGcattaatcatgtcgcaatagcTTTTT |
| shCD36-3-R | AATTAAAAAgctattgcgacatgattaatgCTCGAGcattaatcatgtcgcaatagc |
| miR-539-3p mimic | AUCAUACAAGGACAAUUUCUUU |
| miR-485-3p mimic | GUCAUACACGGCUCUCCUCUCU |
| miR-155-5p mimic | UUAAUGCUAAUCGUGAUAGGGGUU |
| mimic negative control (NC) | UUGUACUACACAAAAGUACUG |
| miR-539-3p inhibitor | AAAGAAAUUGUCCUUGUAUGAU |
| Inhibitor NC | CAGUACUUUUGUGUAGUACAA |

| **Table S2. Western blot antibody information.** | | | |
| --- | --- | --- | --- |
| **Antibody name** | **Catalog#** | **Manufacturers** | **Dilution** |
| P-PI3K | #4228 | Cell Signaling Technology (USA) | 1:500 |
| CD9 | YT0782 | ImmunoWay, China | 1:3000 |
| CD81 | YT5394 |  |  |
| CD63 | YT5525 |  |  |
| Pro-Caspase 3 | ab32499 | Abcam | 1:5000 |
| Bax | 60267-1-Ig | Proteintech, China | 1:5000 |
| Bcl-2 | 68103-1-Ig |  | 1:3000 |
| Cleaved-Caspase 3 | 25128-1-AP |  | 1:2000 |
| CD36 | 18836-1-AP |  | 1:500 |
| PI3K | 20584-1-AP |  |  |
| AKT | 10176-2-AP |  |  |
| P-AKT | 28731-1-AP |  |  |
| β-actin | 20536-1-AP |  | 1:1000 |
| Goat HRP-conjugated anti-Rabbit IgG | SA00001-2 |  |  |
| Goat HRP-conjugated anti-Mouse IgG | SA00001-1 |  |  |

| **Table S3. qPCR primers.** | | |
| --- | --- | --- |
| **Target** | **Forward primer 5’-3’** | **Reverse primer 5’-3’** |
| CD36 | ATGGGCTGTGATCGGAACTG | GTCTTCCCAATAAGCATGTCTCC |
| GLUT2 | TGTTGGGGCCATCAACATGA | AACATGCCAATCATCCCGGT |
| IRS-2 | CGAGTCAATAGCGGAGACCC | CCCCTGAGACCCTACGGTAA |
| NKX6.1 | GGCTGTGGGATGTTAGCTGT | GGCTGTGGGATGTTAGCTGT |
| PDX1 | AGCGTTCCAATACGGACCAG | TGCTCAGCCGTTCTGTTTCT |
| UCP2 | CTGCCAGGACAGTACCCAAG | GACCCGAGTCGCAGAAAAGA |
| 18S | AGGCGCGCAAATTACCCAATCC | GCCCTCCAATTGTTCCTCGTTAAG |
| miR-485-3p | CGCGAGTCATACACGGCTCT | AGTGCAGGGTCCGAGGTATT |
| miR-155-5p | GCGCGTTAATGCTAATTGTGAT |  |
| miR-539-3p | GCGCGCATACAAGGATAATTT |  |
| miR-485-3p reverse transcription primer | GTCGTATCCAGTGCAGGGTCCGAGGTATTCGCACTGGATACGACGAGAGG | |
| miR-155-5p reverse transcription primer | GTCGTATCCAGTGCAGGGTCCGAGGTATTCGCACTGGATACGACACCCCT | |
| miR-539-3p reverse transcription primer | GTCGTATCCAGTGCAGGGTCCGAGGTATTCGCACTGGATACGACAAAAAG | |
| U6 | CTCGCTTCGGCAGCACA | TTTGCGTGTCATCCTTGCG |
| U6 reverse transcription primer | GTCGTATCCAGTGCAGGGTCCGAGGTATTCGCACTGGATACGACAGCGGG | |
