# Supplementary material for: Bone Marrow Mesenchymal Stromal Cells and Their Derived Extracellular Vesicles Protect Pancreatic Beta‐TC‐6 Cells From Hypoxia‐Induced Injury via miR‐539‐3p‐Mediated Downregulation of CD36 Expression
Source: Stem Cells Int. 2026 Jan 21;2026:6616986. doi: 10.1155/sci/6616986 (PMC12824418; doi:10.1155/sci/6616986)

**Supplementary Figure legends**


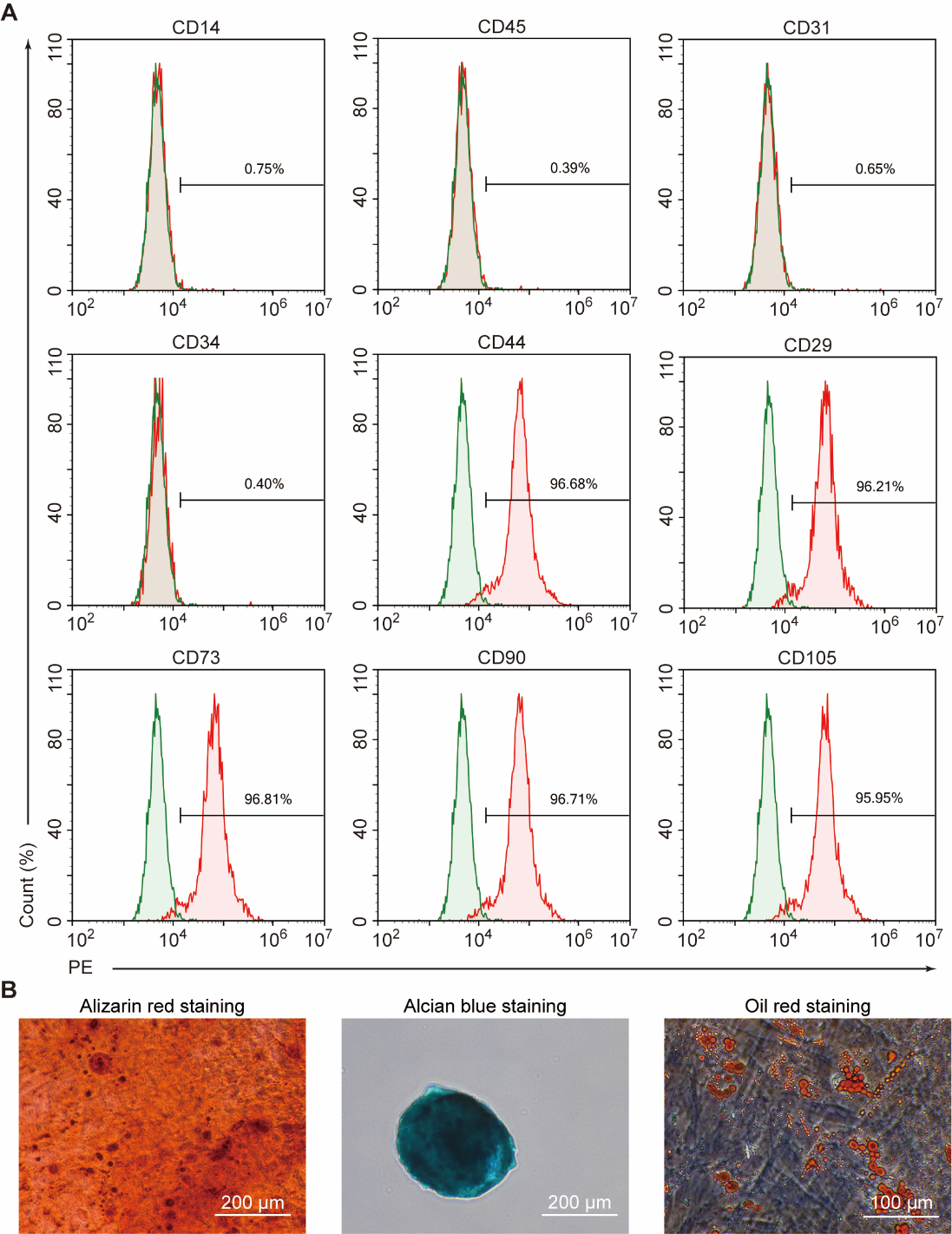


**Figure S1. Characterization of BMSCs.** (A) Flow cytometric analysis results indicating the BMSCs we obtained were positive for CD44, CD29, CD73, CD90, and CD105 and negative for CD14, CD45, CD31, and CD34. (B) Alizarin red, Alcian blue, and Oil red staining results demonstrating the successful differentiation of BMSCs into osteocytes, chondrocytes, or adipocytes.


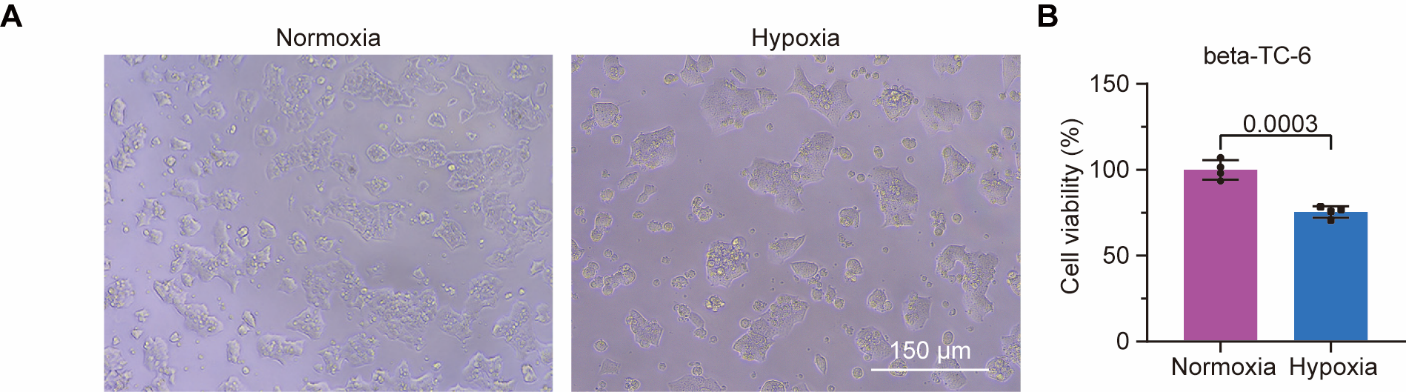


**Figure S2. Establishment of hypoxia cellular model.** (A) Bright-field view images showing the morphology of cells with normoxia (37 °C, 5% CO_2_, 21% O_2_) or hypoxia (37 °C, 5% CO_2_, 2% O_2_) treatment. (B) CCK-8 assay data indicating the cell viability of cells with normoxia or hypoxia treatment.


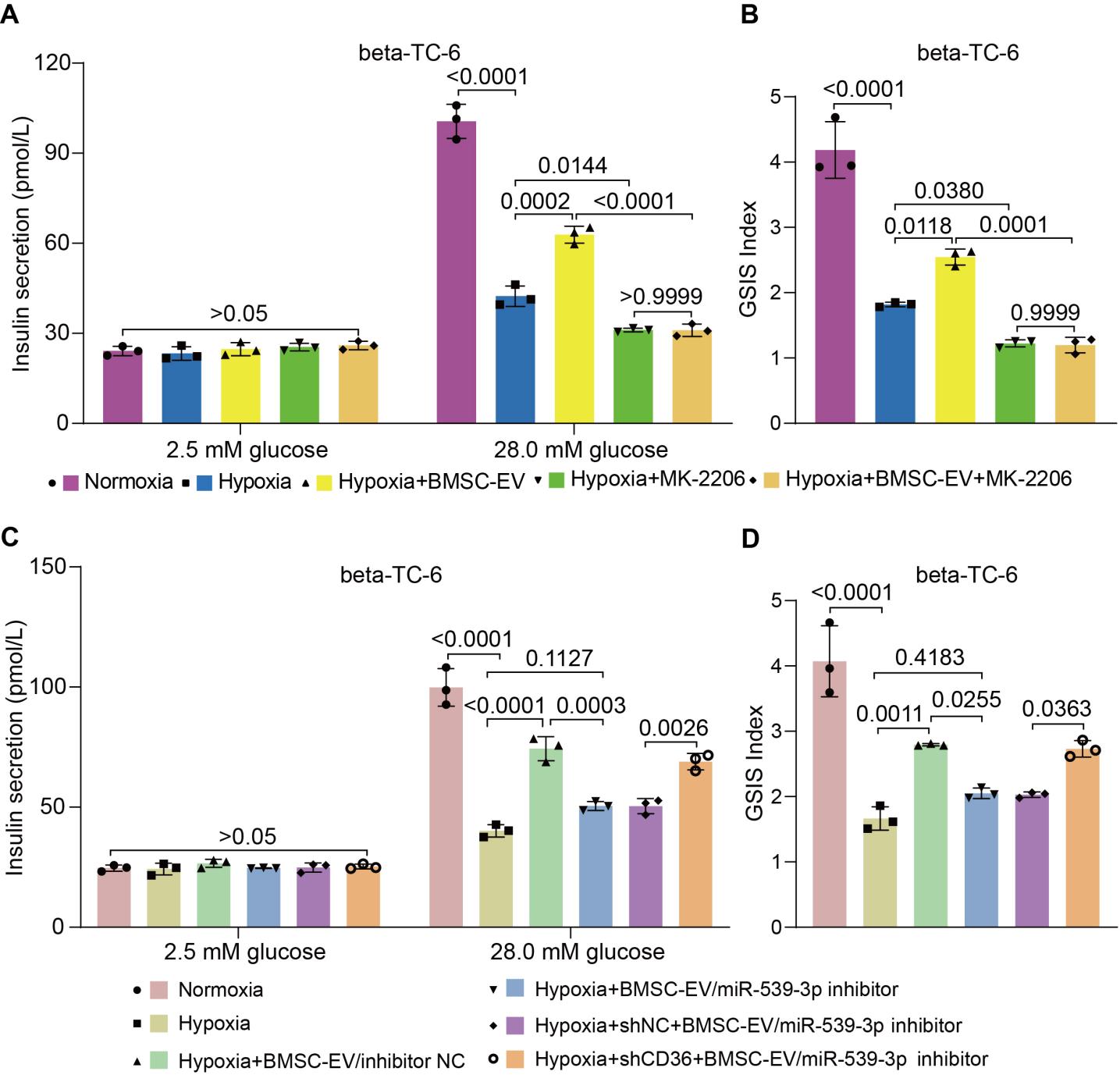


**Figure S3. Detection of insulin secretion levels and glucose stimulated insulin secretion (GSIS) index in beta-TC-6 cells under different treatments.** (A, B) Insulin levels and GSIS index in normoxia, hypoxia, hypoxia+BMSC-EV, hypoxia+MK-2206 and hypoxia+BMSC-EV+MK-2206. (C, D) Insulin levels and GSIS index in normoxia, hypoxia, hypoxia+BMSC-EV/inhibitor NC, hypoxia+BMSC-EV/miR-539-3p inhibitor, hypoxia+shNC+BMSC-EV/miR-539-3p inhibitor and hypoxia+shCD36+BMSC-EV/miR-539-3p inhibitor.


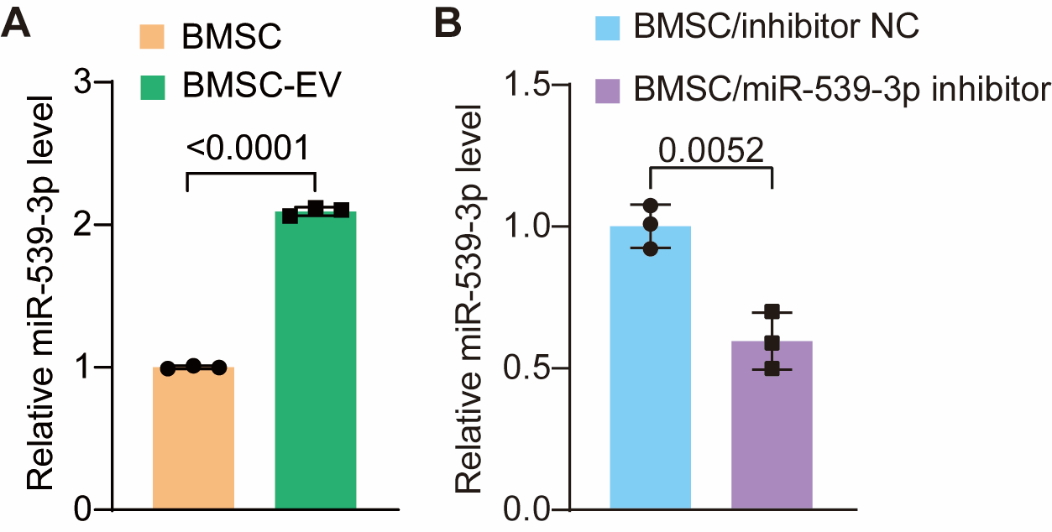


**Figure S4.** **Detection of miR-539-3p level.** (A) qPCR results showing the expression of miR-539-3p in BMSCs and BMSC-EVs. (B) qPCR results showing the expression of miR-539-3p in BMSCs/inhibitor NC and BMSC/miR-539-3p inhibitor.

**
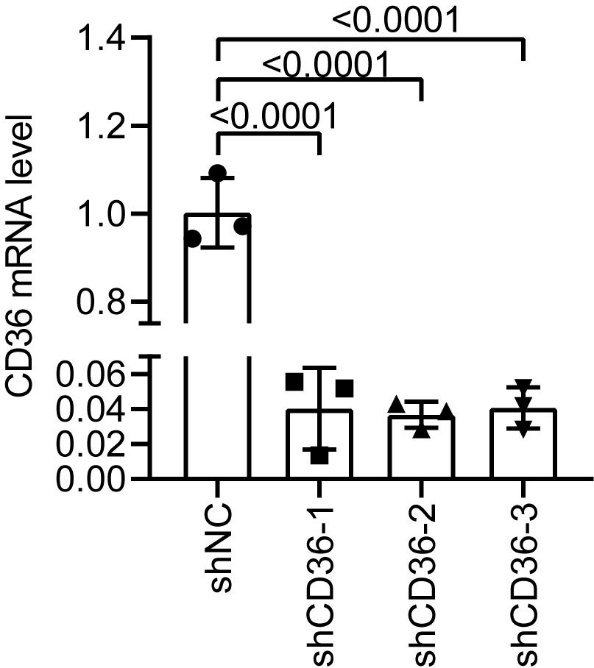
**

**Figure S5.** **Validation of the efficiency of shRNAs targeting CD36.** qPCR results showing the mRNA expression of CD36.

**Figure S6. Full length blots of Western blotting.**

Full length blots of Figure 2A.

**
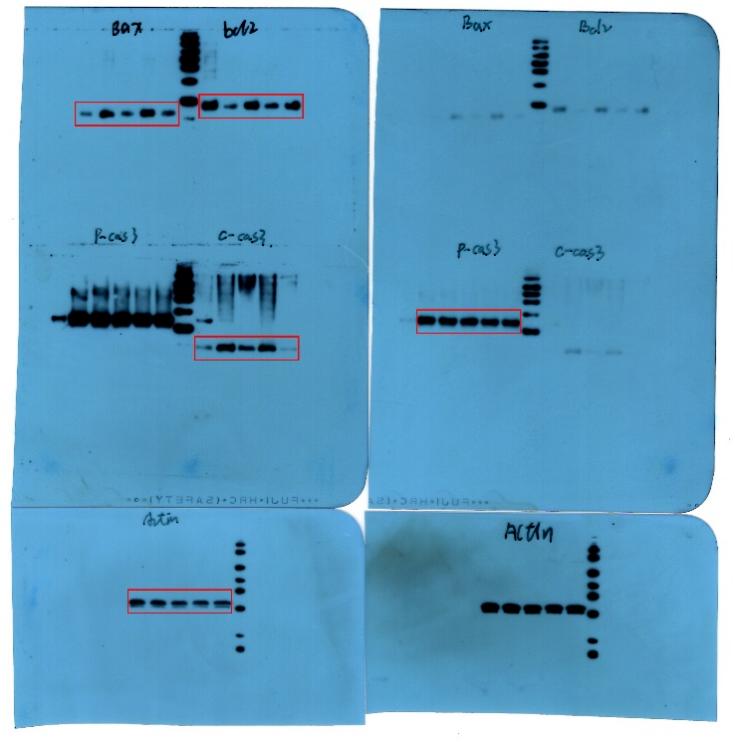
**

Full length blots of Figure 3B.


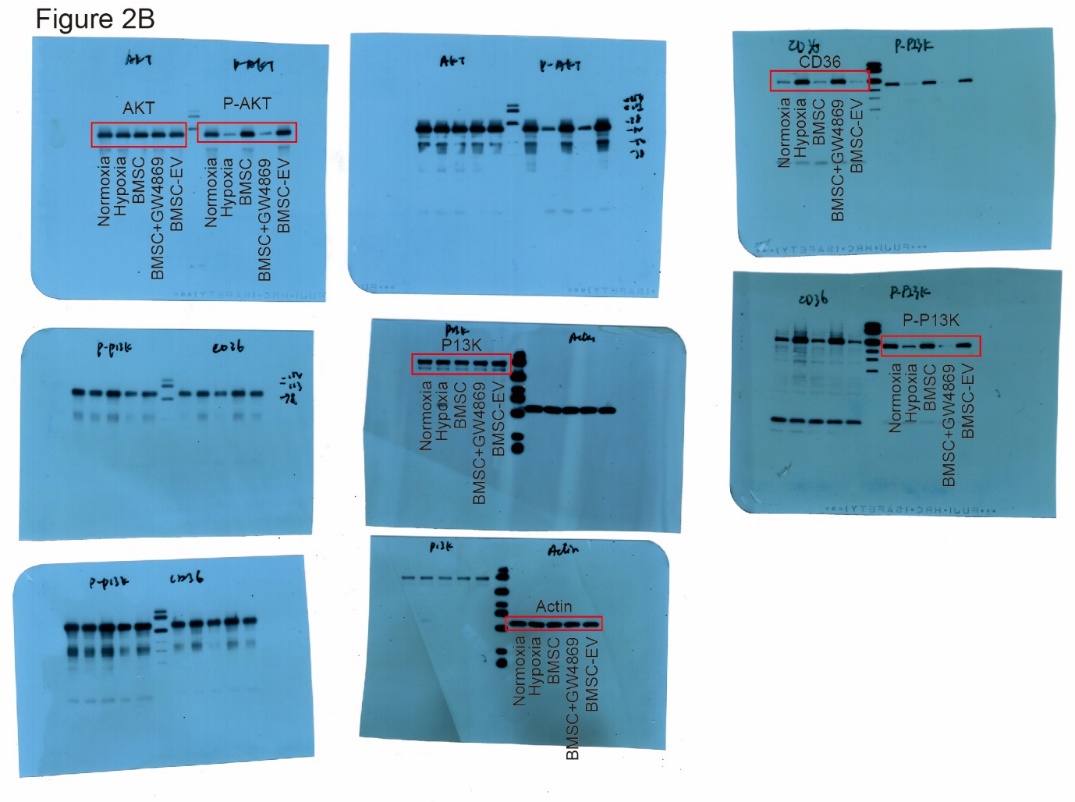


Full length blots of Figure 3D.


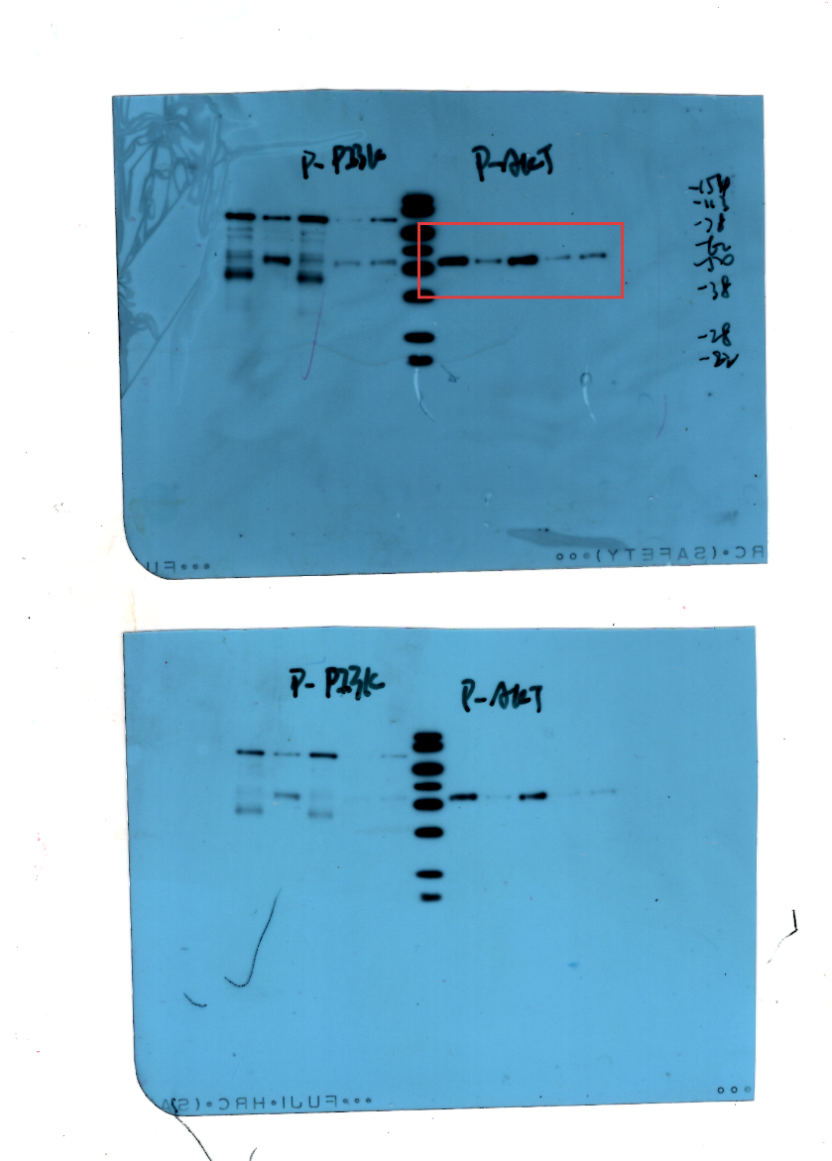

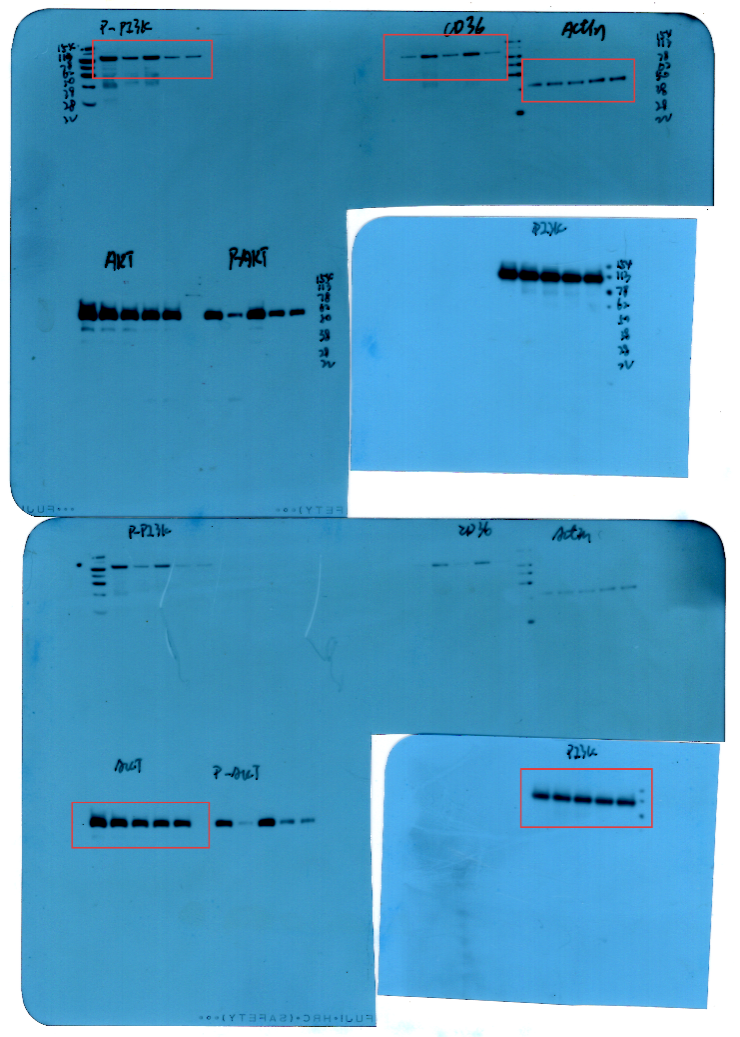


Full length blots of Figure 6C.

**
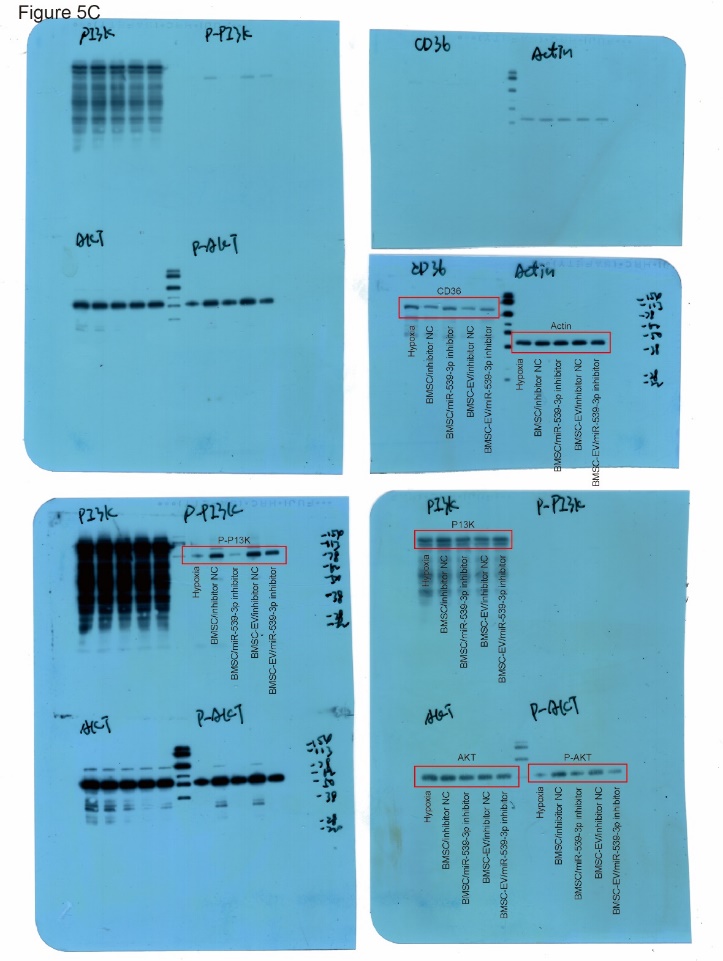
**

Full length blots of Figure 8B.


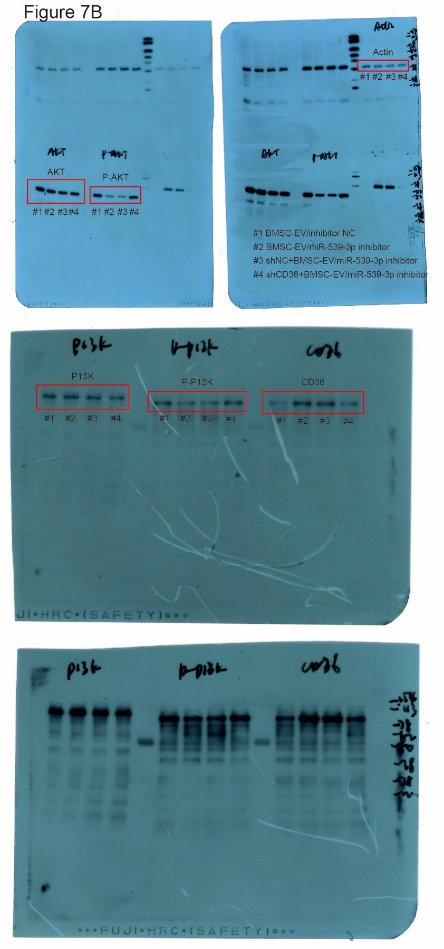

Supplement: Supplementary file 2 — Supporting Information 2 Figure S1: Characterization of BMSCs. Figure S2: Establishment of hypoxia cellular model. Figure S3: Detection of insulin secretion levels and glucose‐stimulated insulin secretion (GSIS) index in beta‐TC‐6 cells under different treatments. Figure S4: Detection of miR‐539‐3p level. Figure S5: Validation of the efficiency of shRNAs targeting CD36. Figure S6: Full‐length blots of western blotting. [file SCI-2026-6616986-s001.docx]
